# Perilipin 2–positive mononuclear phagocytes accumulate in the diabetic retina and promote PPAR**γ**-dependent vasodegeneration

**DOI:** 10.1172/JCI161348

**Published:** 2023-10-02

**Authors:** Guillaume Blot, Rémi Karadayi, Lauriane Przegralek, Thérèse-Marie Sartoris, Hugo Charles-Messance, Sébastien Augustin, Pierre Negrier, Frédéric Blond, Frida Paulina Muñiz-Ruvalcaba, David Rivera-de la Parra, Lucile Vignaud, Aude Couturier, José-Alain Sahel, Niyazi Acar, Aida Jimenez-Corona, Cécile Delarasse, Yonathan Garfias, Florian Sennlaub, Xavier Guillonneau

**Affiliations:** 1 Institute of Vision, Sorbonne University, INSERM, CNRS, Paris, France.; 2 ED394 Physiology and Physiopathology Doctoral School, Sorbonne University, Paris, France.; 3 A. de Rothschild Foundation Hospital, Paris, France.; 4 Comprehensive Care Center for Diabetes Patients, Salvador Zubrian National Institute of Health Sciences and Nutrition, Mexico City, Mexico.; 5 Institute of Ophthalmology “Fundación Conde de Valenciana” I.A.P., Mexico City, Mexico.; 6 Department of Ophthalmology, Hôpital Lariboisière, AP-HP, University of Paris, Paris, France.; 7 Department of Ophthalmology, The University of Pittsburgh School of Medicine, Pittsburgh, Pennsylvania, USA.; 8 CHNO des Quinze-Vingts, Institut Hospitalo-Universitaire FOReSIGHT, INSERM-DGOS CIC 1423, Paris, France.; 9 Eye and Nutrition Research Group, Center for Taste and Food Sciences, CNRS, INRAE, Institut Agro, Bourgogne Franche-Comté University, Dijon, France.; 10 Department of Epidemiology and Visual Health, Instituto de Oftalmología Fundación Conde de Valenciana, Mexico City, Mexico.; 11 General Directorate of Epidemiology, Secretariat of Health, Mexico City, Mexico.; 12 Department of Biochemistry, School of Medicine, National Autonomous University, Mexico City, Mexico.; 13 Cell and Tissue Biology, Research Unit, Instituto de Oftalmología Fundación Conde de Valenciana”, Mexico City, Mexico.

**Keywords:** Inflammation, Ophthalmology, Diabetes, Macrophages, Microcirculation

## Abstract

Type 2 diabetes mellitus (T2DM), characterized by hyperglycemia and dyslipidemia, leads to nonproliferative diabetic retinopathy (NPDR). NPDR is associated with blood-retina barrier disruption, plasma exudates, microvascular degeneration, elevated inflammatory cytokine levels, and monocyte (Mo) infiltration. Whether and how the diabetes-associated changes in plasma lipid and carbohydrate levels modify Mo differentiation remains unknown. Here, we show that mononuclear phagocytes (MPs) in areas of vascular leakage in DR donor retinas expressed perilipin 2 (PLIN2), a marker of intracellular lipid load. Strong upregulation of *PLIN2* was also observed when healthy donor Mos were treated with plasma from patients with T2DM or with palmitate concentrations typical of those found in T2DM plasma, but not under high-glucose conditions. *PLIN2* expression correlated with the expression of other key genes involved in lipid metabolism (*ACADVL*, *PDK4)* and the DR biomarkers *ANGPTL4* and *CXCL8*. Mechanistically, we show that lipid-exposed MPs induced capillary degeneration in ex vivo explants that was inhibited by pharmaceutical inhibition of PPARγ signaling. Our study reveals a mechanism linking dyslipidemia-induced MP polarization to the increased inflammatory cytokine levels and microvascular degeneration that characterize NPDR. This study provides comprehensive insights into the glycemia-independent activation of Mos in T2DM and identifies MP PPARγ as a target for inhibition of lipid-activated MPs in DR.

## Introduction

Type 2 diabetes mellitus (T2DM) affects a growing part of the population worldwide (1). Fifteen years after the onset of diabetes, more than 60% of patients will develop some form of diabetic retinopathy (DR) (2). DR stages are clinically characterized by the observation of retinal vascular damage (3). Alterations in the blood-retina barrier and the resulting progressive degeneration of retinal capillaries, ensuing retinal ischemia, extravasation, and bleeding occur in nonproliferative DR (NPDR), the early stage of the disease. Severe NPDR can ultimately progress to sight-threatening proliferative DR (PDR), which is characterized by the formation of neovascular tufts around the ischemic retinal tissue. These areas of vascular remodeling are associated with neurodegenerative processes and innate immune dysregulation (4, 5). High intraocular levels of inflammatory cytokines are found in patients with DR, and cytokines such as TNF-α, IL-1β, IL-6, and IL-8 (CXCL8) have been proposed to be involved in the progression of DR (5, 6). Consistent with such a role for cytokines, treatments targeting inflammation have been shown to be protective against diabetes-induced neurodegeneration and vascular remodeling (7). Activation of retinal mononuclear phagocytes (MPs) has been described in patients with diabetes (8) and in animal models of T2DM (9, 10). The retinal MP population in DR is composed not only of resident microglia but also of infiltrating monocyte-derived (Mo-derived) macrophages. The latter play a prominent role in inflammation and vascular remodeling in DR (11–14).

DR is a multifactorial disease, and hyperglycemia only accounts for a limited proportion of the risk of developing DR (15). Indeed, a number of other glycemic-independent risk factors are associated with the development and progression of DR (16–18).

Dyslipidemia has been a largely neglected field of study but has recently attracted attention and is now considered to be an important area of research in DR (17, 18). Indeed, numerous studies (19–25), as well as a meta-analysis (26), have demonstrated correlations between plasma lipid levels and DR or diabetic edema in the past 10 years. Moreover, the lipid-lowering drug fenofibrate was shown to slow DR progression independently of glycemic control in 2 large randomized, controlled clinical trials (27, 28). Saturated free fatty acids (FFAs) are generally considered to be proinflammatory, whereas unsaturated FFAs are classically considered to be antiinflammatory (29–32). Palmitate (PA) (saturated 16-carbon FA [C16:0]) is the most abundant FFA in the plasma (33), and its concentration is found to be elevated among patients with T2DM (34, 35). MPs can take up and store lipids in intracellular lipid droplets stabilized by the amphiphilic protein perilipin 2 (PLIN2) (36). PLIN2^+^ lipid-laden MPs (PLIN2^+^ MPs) are found in atherosclerotic lesions and in fat lesions after trauma (37). In the brain, PLIN2^+^ MPs were found in grade 2 cerebellar infarcts, whereas they were absent in control and low-grade infarcts (37). More recently, lipid-laden MPs were found to progressively accumulate in the aging mouse and human brain (38). PLIN2^+^ MPs exhibit alterations in their metabolism, phagocytosis, and autophagy, leading to the production of ROS and inflammatory cytokines (38–42). The presence and role of MPs exposed to plasma lipids in the retina in DR remain to be determined.

In the present study, MPs expressing PLIN2 were found in the retinas of patients with DR in regions with active vascular leakage and vascular remodeling. Next, we aimed to understand the molecular cues that drive Mos to differentiate into PLIN2^+^ MPs and their role in vascular remodeling. We show that exposure of naive human Mos to heat-inactivated plasma from patients with diabetes or to FFAs was sufficient to induce *PLIN2* expression and secretion of DR-associated inflammatory cytokines. Experimentally, we show that lipid-exposed MPs had strong vasodegenerative properties that were inhibited by a pharmacological PPARγ inhibitor. Overall, these results pave the way for potential treatment targeting MP PPARγ signaling in dyslipidemia-mediated inflammation, such as DR, and potentially beyond retinal vasodegeneration.

## Results

### Human diabetic retina postmortem analysis reveals PLIN2^+^ MP accumulation in regions with vascular leakage.

**Figure 1:**
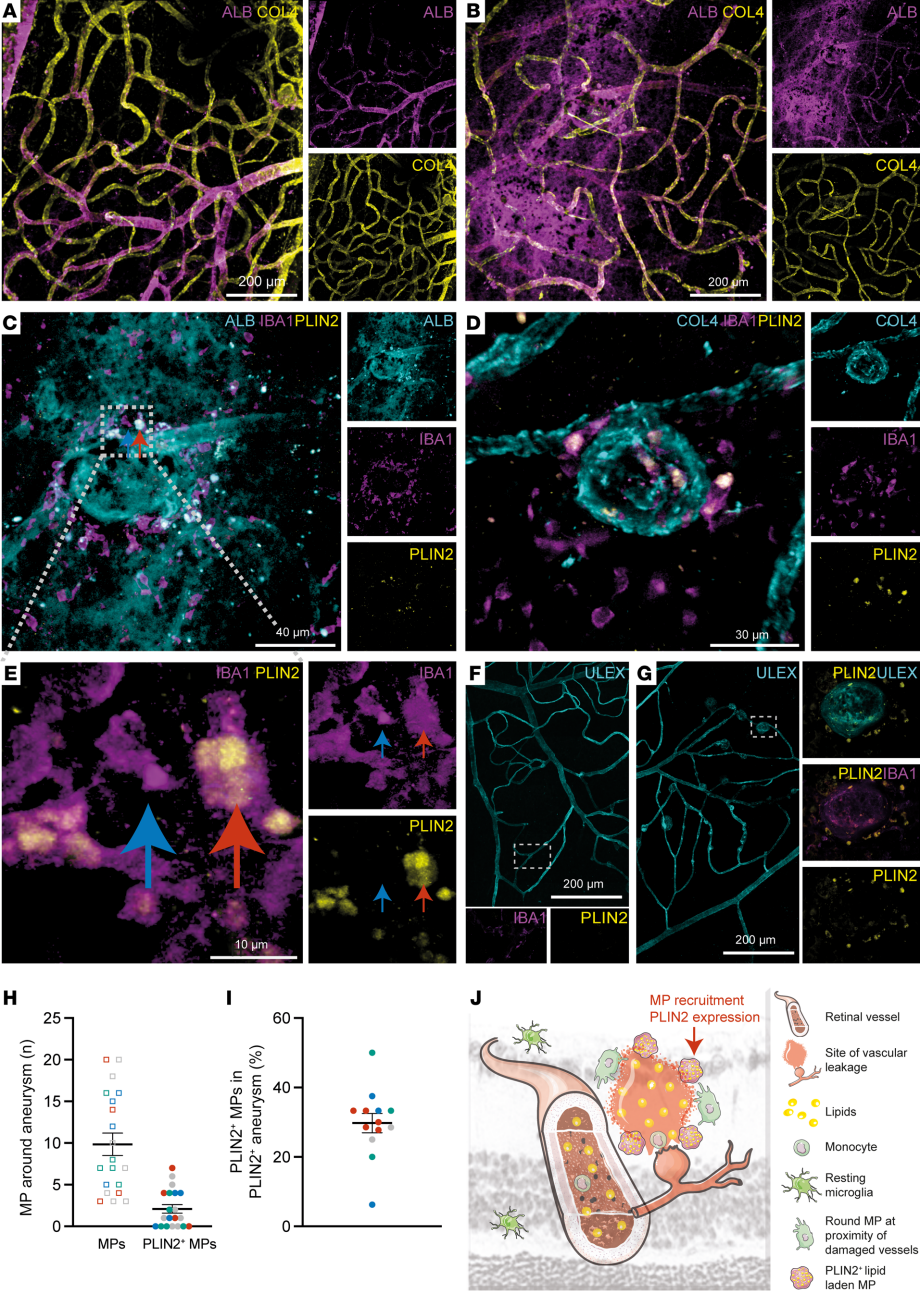
PLIN2^+^ MPs are found in regions with retinal plasma leakage in a patient with DR. (**A**–**G**) Representative immunofluorescence (IF) images of human flat-mounted retinas from 4 postmortem DM donors. (**A**) IF staining of ALB (plasma protein) and COL4 (vessels) within a healthy vasculature area of human retina. Scale bar: 200 μm. (**B**) IF staining for ALB and COL4 within an area showing vascular leakage. Scale bar: 200 μm. (**C**) IF staining for ALB, IBA1, and PLIN2 within an area with a leaking microaneurysm. Scale bar: 40 μm. (**D**) IF staining for COL4, IBA1 (MPs), and PLIN2 (neutral lipid droplets) within an area with a microaneurysm. Scale bar: 30 μm. (**E** and **F**) IF staining for IBA1 and PLIN2 and labeling with UEA1 (vessels). (**E**) IF staining for IBA1 and PLIN2. The arrows show a PLIN2^–^ (blue) and a PLIN2^+^ (red) MP within the same region. Scale bar: 10 μm. (**F**) Representative image outside the area with the microaneurysms. Scale bar: 200 μm. (**G**) Representative image within the area of the microaneurysms. (**H**) Total MP count and PLIN2^+^ MP count around microaneurysms in 4 different DM donors (each color represents a different donor). (**I**) Percentage of PLIN2^+^ MPs within microaneurysms containing at least 1 PLIN2^+^ MP. (**J**) Schematic depiction of a ruptured retinal microaneurysm leading to plasma leakage in a DR retina.

PLIN2^+^ MPs have been shown to progressively accumulate in the brains of aging mice and humans and in regions of cerebellar infarcts. Lipid-laden macrophages have been reported in the retina following blood-retina barrier breakdown (43), but the localization of PLIN2^+^ MPs in the diabetic retina has not been reported thus far. We identified regions with potential PLIN2^+^ cells by staining retinas from a collection of postmortem diabetic donor retinas (Supplemental Table 1; supplemental material available online with this article; https://doi.org/10.1172/JCI161348DS1) with anti–collagen IV (COL4), anti-albumin (anti-ALB), anti-IBA1, a pan-MP antibody, and anti-PLIN2 antibodies. COL4 and ALB staining revealed regions where ALB was found only in the vessel lumen (Figure 1A) and regions with microaneurysms where extraluminal ALB was found (Figure 1B). IBA1 staining identified numerous MPs in contact with retinal aneurysms, and 2 anti-PLIN2–stained samples showed identical localization and cellular patterns (Figure 1, C–F). All PLIN2^+^ cells were also stained with IBA1 and ALB, which clearly identified PLIN2^+^ cells as intraretinal MPs (Figure 1, C–F) that had taken up plasma leakage material. Higher magnification showed the presence of both PLIN2^+^ and PLIN2^–^ MPs at the sites of the aneurysm (Figure 1E). To determine the proportion of PLIN2^+^ MPs in retinal tissue obtained from postmortem donors, we used anti-IBA1 and anti-PLIN2 antibodies to stain retinal MPs and Ulex europaeus I agglutinin (UEA1, a marker of human vascular endothelial cells) to costain vessels (Figure 1, F and G). No PLIN2^+^ cells were observed in regions without microaneurysms in the diabetes mellitus (DM) retina (Figure 1F), nor were these cells found in the control patient’s retina (data not shown). We then counted the number of MPs that fell into each category in large-field retinal flat-mount images from the 4 different postmortem DM donors (Figure 1, G–I). In the diseased retina, we detected between 4 and 21 aneurysms per field. We analyzed a minimum of 4 aneurysms and up to 7 per DM donor for a total of 19 aneurysms analyzed. On average, we observed 10 MPs per aneurysm, 2 of which were PLIN2^+^. In detail, we found 7 aneurysms that were exclusively composed of PLIN2^–^ MPs (Figure 1H). The remaining 12 aneurysms (63.2%) were made up of 29.8% PLIN2^+^ MPs (Figure 1I). Similar to other reports on the aging or infarcted brain, PLIN2^+^ MPs were found in the vicinity of damaged capillaries in human samples of DR retinas (Figure 1J).

### PLIN2 expression in PA-exposed MPs is associated with changes in lipid metabolism genes and expression of key DR markers.

**Figure 2:**
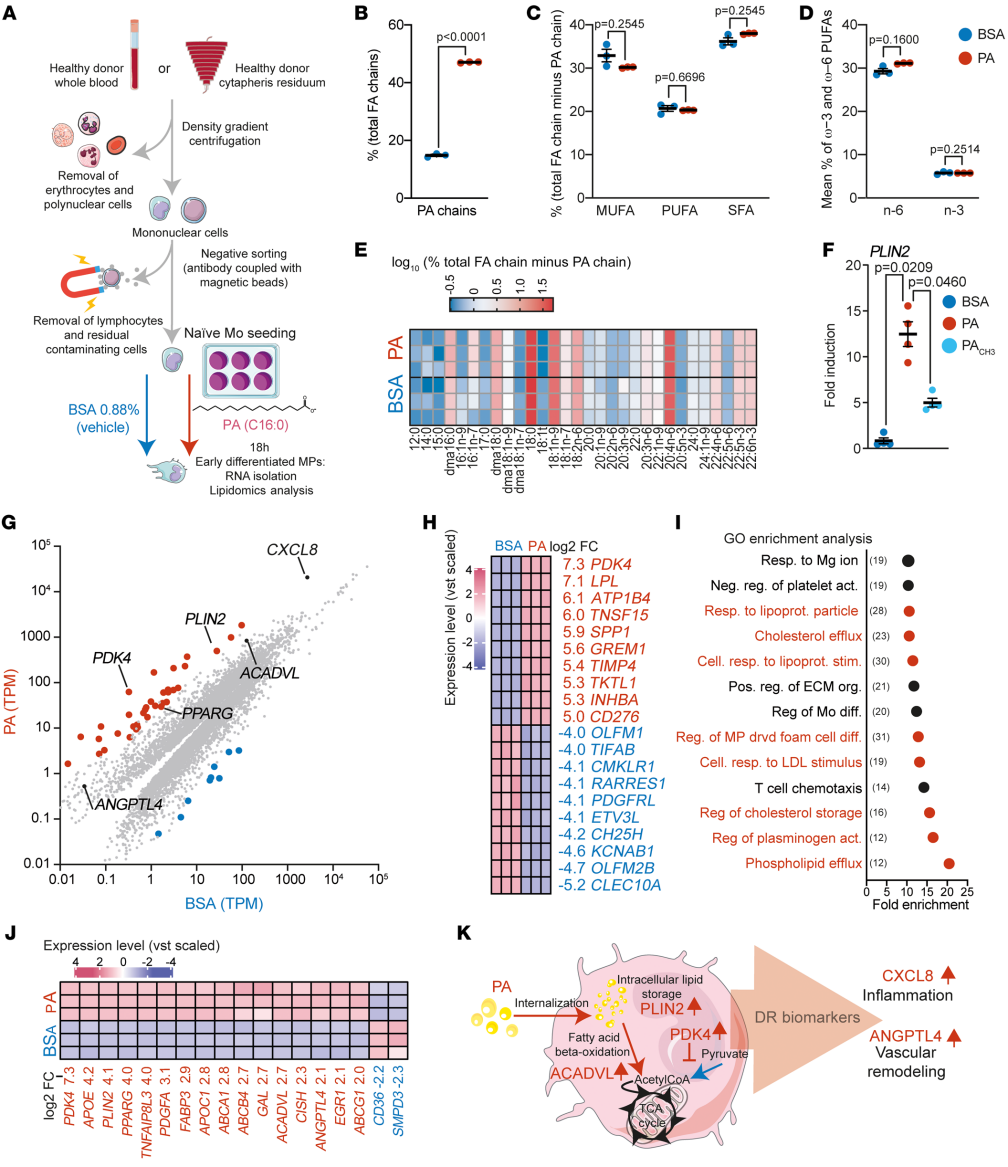
PA stimulation triggers the expression of key DR markers by MPs. (**A**) Schematic representation of Mo isolation, treatment, and preparation. (**B**–**D**) Chromatographic analysis of FA chain composition after BSA (*n* = 3) or PA (*n* = 3) treatment. (**B**) Mean percentage of PA (C16:0) chains relative to total FAs. *P* s were calculated using Welch’s *t* test. (**C**) Mean percentage of monounsaturated FAs (MUFAs), polyunsaturated FAs (PUFAs), and saturated FAs (SFAs) (minus PA). (**D**) Mean percentage of ω−3 and ω–6 PUFAs. (**C** and **D**) *P* values were calculated using multiple Welch’s *t* tests corrected for multiple comparisons using the Holm-Šidák method. (**E**) Heatmap representation of the percentage of individual FA chains relative to total FA chains (minus PA). (**F**) RT-qPCR quantification of *PLIN2* after treatment with BSA (*n* = 4), PA (*n* = 4), or PA_CH3_ (*n* = 4). *P* values were determined by 1-way Welch’s ANOVA (*P* = 0.0021) followed by Dunnett’s T3 multiple-comparison test. (**G**–**I**) RNA-Seq transcriptomics analysis after BSA (*n* = 3) or PA (*n* = 3) treatment. (**G**) Scatter plot of the mean TPM value for all transcripts detected after BSA (*x* axis) or PA (*y* axis) treatment. The red and blue dots represent transcripts upregulated with a log_2_ FC of 4 or higher or a log_2_ FC of 4 or lower. (**H**) Heatmap representation of the log_2_ variance stabilizing transformation (vst) of the top 10 upregulated and downregulated transcripts. (**I**) GO enrichment analysis representing the fold enrichment of the 528 transcripts with a log_2_ FC of 2 or higher; red dots represent pathways related to lipid metabolism. Numbers in parenthesis represents the number of genes regulated by PA stimulation. Resp., response; Neg., negative; reg., regulator; act. activation; lipoprot., lipoprotein; Cell., cellular; stim., stimulation; Pos., positive; org., organization; diff., differentiation; drvd, derived. (**J**) Heatmap representation of the log_2_ vst of transcripts with a log_2_ FC of 4 or higher and belonging to the GO pathway “fatty acid metabolic process.” (**K**) Schematic representation of the biological function of the markers selected as a signature of lipid exposure.

NPDR is characterized by blood-retina barrier breakdown, Mo infiltration, and plasma extravasation into the retina. Our PLIN2 immunohistochemical studies (Figure 1) suggested that resident MPs and/or infiltrating Mos had taken up extravasated lipids. We investigated the molecular mechanism by which plasma lipids drive the differentiation of Mos into lipid-associated MPs (PLIN2^+^ MPs) by exposing naive Mos to a blood-mimicking (500 μM) concentration of PA (34). We first compared the cellular FA chain composition in early Mo-derived MPs 18 hours after exposure of naive Mos to PA bound to BSA or BSA alone (Figure 2A). Lipidomic analysis showed that PA was rapidly internalized by differentiating Mos and represented 47.9% of the total FA chains in PA-treated, early-differentiated MPs, whereas it represented only 14.7% of the FA in the BSA-only condition (Figure 2B). PA stimulation did not affect the relative abundance of total saturated (other than PA), monounsaturated, total polyunsaturated, or total omega-3 or total omega-6 polyunsaturated FAs (Figure 2, C and D) or induce a change in the composition of individual non-PA FA chains (Figure 2E and Supplemental Table 2). Concomitant with PA influx, *PLIN2* expression significantly increased in MPs, whereas the addition of methyl-PA (PA_CH3_), a PA analog that can be internalized but that is less biologically active and not readily metabolizable, resulted in partial inhibition of this induction (Figure 2F). Overall, our results suggest that naive Mos actively adapted their transcriptomic profile in response to PA exposure. How Mos and early Mo-derived MPs adapt to a lipid-rich microenvironment is unknown. We aimed to obtain an unbiased view of the transcriptomic changes induced by a lipid-rich environment during differentiation. We thus exposed naive human Mos from healthy donors to PA (500 μM) or to BSA (Figure 2A) for 18 hours, a time point representative of early Mo differentiation (44), and compared their transcript expression levels by RNA-Seq. The expression level, in transcripts per million (TPM), of each transcript in each condition was determined (Supplemental Table 3), and the means are presented as scatter plots for the BSA condition (*x* axis) and the PA condition (*y* axis) (Figure 2G). DESeq2 analysis showed that PA treatment induced a statistically significant (adjusted *P* ≤ 0.05) differential expression of 9,794 transcripts (Supplemental Table 3). Among them, 528 (5.4 %) were regulated with a log_2_ fold change (FC) of greater than 2, of which 37 transcripts (0.4%, red dots) were upregulated more than 16-fold (Figure 2G). The top 10 up- and downregulated transcripts are shown in Figure 2H. We then performed a gene ontology (GO) enrichment analysis of these 528 regulated transcripts to determine the major functions affected by PA treatment. Thirteen GO terms were significantly enriched, 8 of which were related to lipid metabolism (red dots), consistent with the adaptation of Mo metabolism to the lipid-rich environment (Figure 2I). This observation was further confirmed by specific analysis of the broad GO term “fatty acid metabolic process” (414 genes). Among them, 229 of the 414 transcripts (55.3%) were found to be differentially expressed following PA versus BSA treatment. Sixteen (7.8%) were among the 37 highly regulated genes (FC ≥4) (Figure 2J and Supplemental Table 4). Only 2 were downregulated (*SMPD3* and *CD36*; log_2_ FCs = –2.29 and –2.22, respectively) (Figure 2H and Supplemental Table 4). Our analysis confirmed the upregulation of *PLIN2* and identified *CXCL8* as the third most highly expressed transcript in PA-stimulated Mos, with a log_2_ FC of 2.87 (Figure 2, G and J). PA stimulation also induced a strong upregulation of *ANGPTL4* (log_2_ FC = 2.14). ANGPTL4 acts as an endogenous inhibitor of lipoprotein lipase (LPL), an enzyme responsible for breaking down triglycerides (TGs) in circulating lipoproteins, and its levels in aqueous humor have been shown to correlate with areas of nonperfusion in patients with DR (45, 46).

Overall, our transcriptomic analysis showed a modification in lipid metabolism and DR-related cytokine expression in Mos in response to PA. We chose 4 markers that are highly regulated by PA exposure for downstream analysis: *PLIN2* (for its role in intracellular lipid droplet stabilization, log_2_ FC = 4.13), *PDK4* (an inhibitor of pyruvate conversion into acetyl-CoA, which was the most highly regulated transcript by PA treatment, log_2_ FC = 7.27), *ACADVL* (for its role in lipid β-oxidation, log_2_ FC = 2.7), and *ANGPTL4* (for its role in regulating TG uptake and its involvement in DR physiopathology, log_2_ FC = 2.14) (Figure 2K). To ensure that our results were not biased by the ability of BSA to bind FFAs released by the cells, we also performed RNA-Seq analysis of gene expression in naive Mos stimulated with PA in the presence of BSA, compared with a control medium without BSA. *PLIN2*, *PDK4*, *ACADVL*, *ANGPTL4*, and *CXCL8* were also upregulated when MPs grown in BSA-free conditions were used as controls, and a very similar regulation of the genes involved in the “fatty acid metabolic process” was also observed (Supplemental Figure 1).

We then studied the effect on MP polarization of other FFA species that are also found to be elevated in patients with T2DM, such as stearate (SA) (C18:0), the second most abundant saturated FFA in the blood, and palmitoleate (PoA) (C16:1 n-7), an unsaturated form of PA. SA and PoA also demonstrated the ability to upregulate *PLIN2*, *PDK4*, *ACADVL*, *ANGPTL4*, and *CXCL8* transcripts (Supplemental Figure 2A) (34, 35).

To gain a deeper understanding of the influence of FFA mixtures on MP polarization, we implemented an expanded set of stimulations. We then investigated the potential modulatory role of oleic acid (OA) (C18:1 n-9), one of the most abundant circulating FAs traditionally considered to be antiinflammatory, in PA stimulation. Naive Mos were stimulated with an equimolar mixture of PA and OA (250 μM each, totaling 500 μM FFA), and transcript expression was compared with that induced by 500 μM PA alone. Interestingly, we found that the equimolar mixture significantly increased the expression of *PLIN2* and *PDK4* compared with expression following stimulation with PA alone, whereas, in sharp contrast, *CXCL8* was strongly downregulated. However, despite the presence of OA, we still observed overexpression of our selected markers *PLIN2*, *PDK4*, *ACADVL*, *ANGPTL4*, and *CXCL8* (Supplemental Figure 2B).

Considering the complexity of the FFA milieu in vivo, we developed a custom FFA blend with a target concentration of 500 μM to allow a meaningful comparison with standard PA stimulation. According to Yi et al., the 4 most abundant FAs in the blood are PA (136 μM), SA (46 μM), OA (117 μM), and linoleic acid (LA) (132 μM), collectively constituting 84% (430 μM) of the total FAs (509 μM) (34). The custom blend of these 4 major FAs was therefore formulated as follows to mimic their relative abundance in the bloodstream: PA, 157 μM; SA, 53 μM; OA, 136 μM; and LA, 153 μM (as detailed in Supplemental Table 5). As with the PA-OA equimolar mixture, this plasma representative blend also induced a trend toward higher expression of *PLIN2*, *PDK4*, and *ACADVL* (although these changes were statistically significant only for *PDK4*) when compared with 500 μM PA alone. Interestingly, *ANGPTL4* was found to be significantly downregulated after FFA blend stimulation. Consistent with what we observed with the PA-OA equimolar mixture, the FA mixture also induced an overexpression of all of our selected markers (*PLIN2*, *PDK4*, *ACADVL*, *ANGPTL4*, and *CXCL8*) compared with the BSA condition (Supplemental Figure 2B).

Last, we replaced PA with an unreadily metabolizable form of PA, PA_CH3_, in the blend. Under these conditions, we observed a nonstatistically significant trend toward lower expression of lipid-related transcripts, which correlated with a statistically significant reduction in expression of the inflammatory cytokine *CXCL8* (Supplemental Figure 2B).

### Stimulation of naive Mos with T2DM plasma increases the expression of PLIN2, which is correlated with the expression of lipid metabolism and DR marker genes.

**Figure 3:**
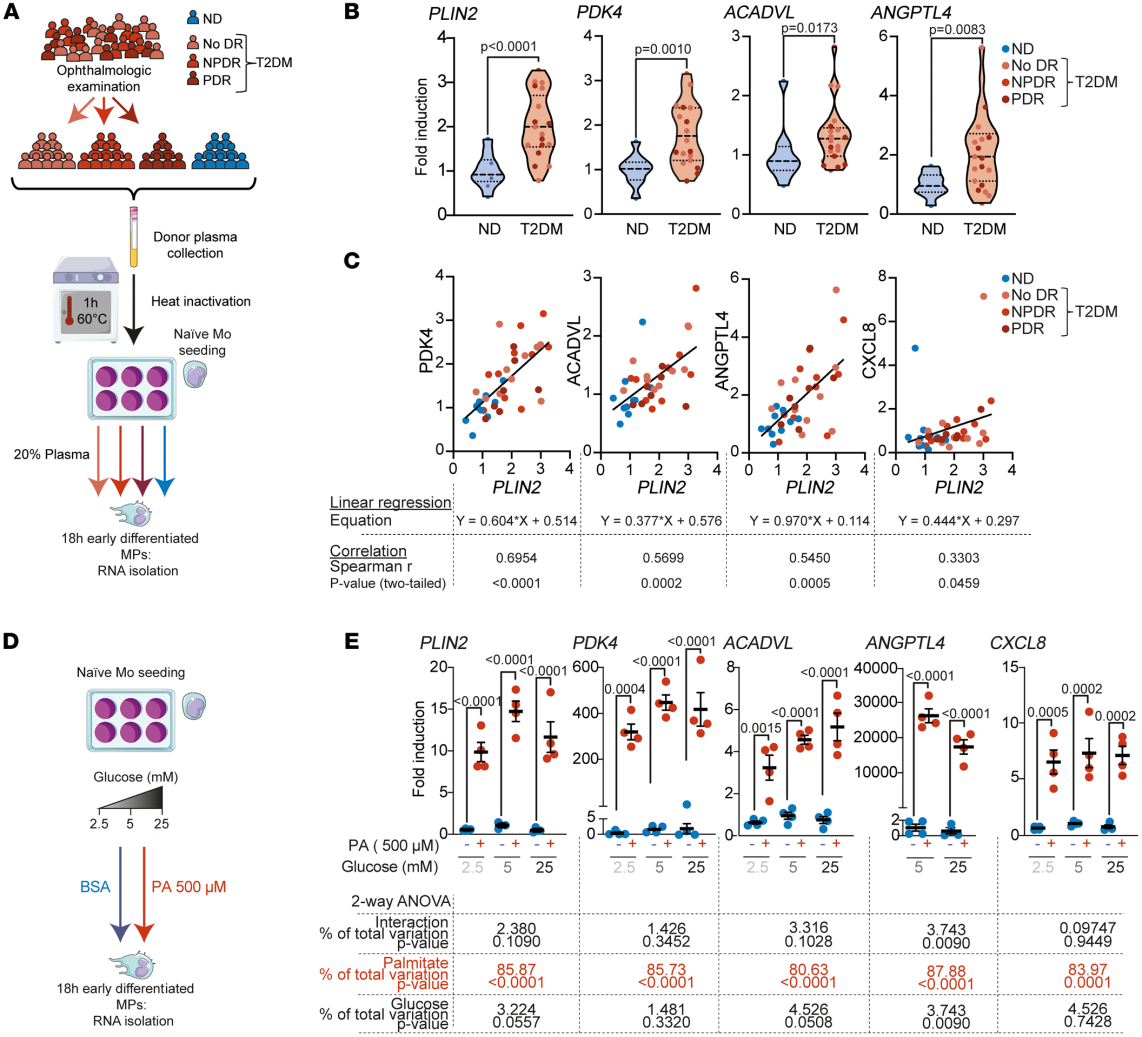
T2DM plasma, but not glucose, induces a lipid-associated phenotype in MPs. (**A**) Schematic representation of donor phenotyping and group attribution, plasma preparation, and naive Mo treatment. (**B** and **C**) RT-qPCR quantification of healthy donor naive Mos treated for 18 hours with donor plasma from ND individuals (*n* = 10 [blue dots]) or patients with T2DM (*n* = 27, no DR [light pink dots], NPDR [pink dots], PDR [dark red dots]). (**B**) Violin plot representation of the relative expression of the indicated genes in response to individual donor plasma exposure. Dashed lines represent the median and quartiles. *P* values were determined using a 2-tailed Mann-Whitney *U* test. (**C**) Simple linear regression representation of *PDK4*, *ACADVL*, *ANGPTL4,* and *CXCL8* (*y* axis) and *PLIN2* expression (*x* axis). Correlations between expression levels were analyzed using Spearman’s correlation; the linear regression equation, Spearman’s *r* [95% CI], and 2-tailed *P* values are given below each correlation graph. (**D**) Schematic representation of naive Mo treatment with PA and increasing concentrations of glucose. (**E**) Scatter plot representation of RT-qPCR expression of selected markers in healthy donor naive Mos treated for 18 hours (or 42 hours for *ANGPTL4*) with either BSA (unbound BSA, blue dots) or PA (BSA-bound PA, red dots) and various concentrations of glucose. Values represent the mean ± SEM of 4 independent cultures. Statistical differences were analyzed by 2-way ANOVA interaction, and *P* values for the PA and glucose treatments are given below each graph.

We next analyzed the expression of the key markers defined above in naive Mos exposed to plasma from patients with diabetes. We established a cohort of donors (Table 1 and Figure 3A) consisting of control nondiabetic (ND) donors (*n* = 10) and diabetic patients with no signs of retinopathy (T2DM no DR, *n* = 10), early-to-mild NPDR (T2DM NPDR, *n* = 10), or PDR (T2DM PDR, *n* = 7) and collected plasma from each (total: *n* = 10 ND donors and *n* = 27 patients with T2DM). The mean leukocyte count and plasma creatinine concentration were similar between the control and T2DM groups (Table 1). The patients with TD2M (*n* = 27) had been diagnosed with T2DM for a median duration of 15 years, and their median age was also higher than that of the ND donors (63 vs. 50 years) (Table 1). The median glycemia and glycated hemoglobin levels for the T2DM donors were 185 mg/dL and 8.4%, respectively, and 85 mg/dL and 4.85%, respectively, for the ND donors. The same purification of naive Mos from a healthy human donor was sampled and exposed for 18 hours to 20% heat-inactivated plasma from each of the 37 donors in the cohort. Heat inactivation for 1 hour at 60°C (hi) was performed to denature and inactivate endogenous plasma cytokines that could have interfered with the Mo and cytokine measurements (47) (Figure 2A). Reverse transcription quantitative PCR (RT-qPCR) analysis showed that expression of the PA-inducible transcripts *PLIN2*, *PDK4*, *ACADVL*, and *ANGPTL4* (Figure 2) was significantly higher in Mos exposed to T2DM patients’ hi-plasma than in those exposed to ND donor hi-plasma (Figure 3B). Although our study did not reveal significant differences between the T2DM subgroups (Supplemental Figure 3), we found a highly significant correlation between the expression of *PDK4*, *ACADVL*, *ANGPTL4*, and *CXCL8* and of *PLIN2* in the entire T2DM group (Figure 3C). This finding links the expression of lipid overload markers, such as *PLIN2*, in early differentiating Mos with known DR-associated cytokines, such as *ANGPTL4* and *CXCL8* (37, 45).

As glycemia also differs between T2DM and ND plasma (34, 35), we next tested the effect of 2.5 mM, 5 mM, and 25 mM glucose in the presence or absence of PA on the differentiation of naive Mos (Figure 3D). The expression of *PLIN2*, *PDK4*, *ACADVL*, and *CXCL8* was again induced by the presence of PA but was not altered by increasing glucose concentrations, and a high glucose concentration did not potentiate the effect of PA (Figure 3E). *ANGPTL4* was only weakly expressed in Mos after 18 hours of PA stimulation but was highly expressed at 42 hours. As for the other transcripts, *ANGPTL4* expression was not potentiated by high glucose (Figure 3E).

In summary, our results show that heat-resistant components of T2DM plasma induce an expression pattern in human Mos very similar to that seen with PA. The lack of induction by glucose and the strong induction of lipid overload marker expression by the T2DM plasma strongly suggest that elevated plasma levels of FFA, such as PA, are responsible for the T2DM-induced shift in Mo polarization.

### Dose-dependent and long-term effects of PA stimulation on naive Mos.

**Figure 4:**
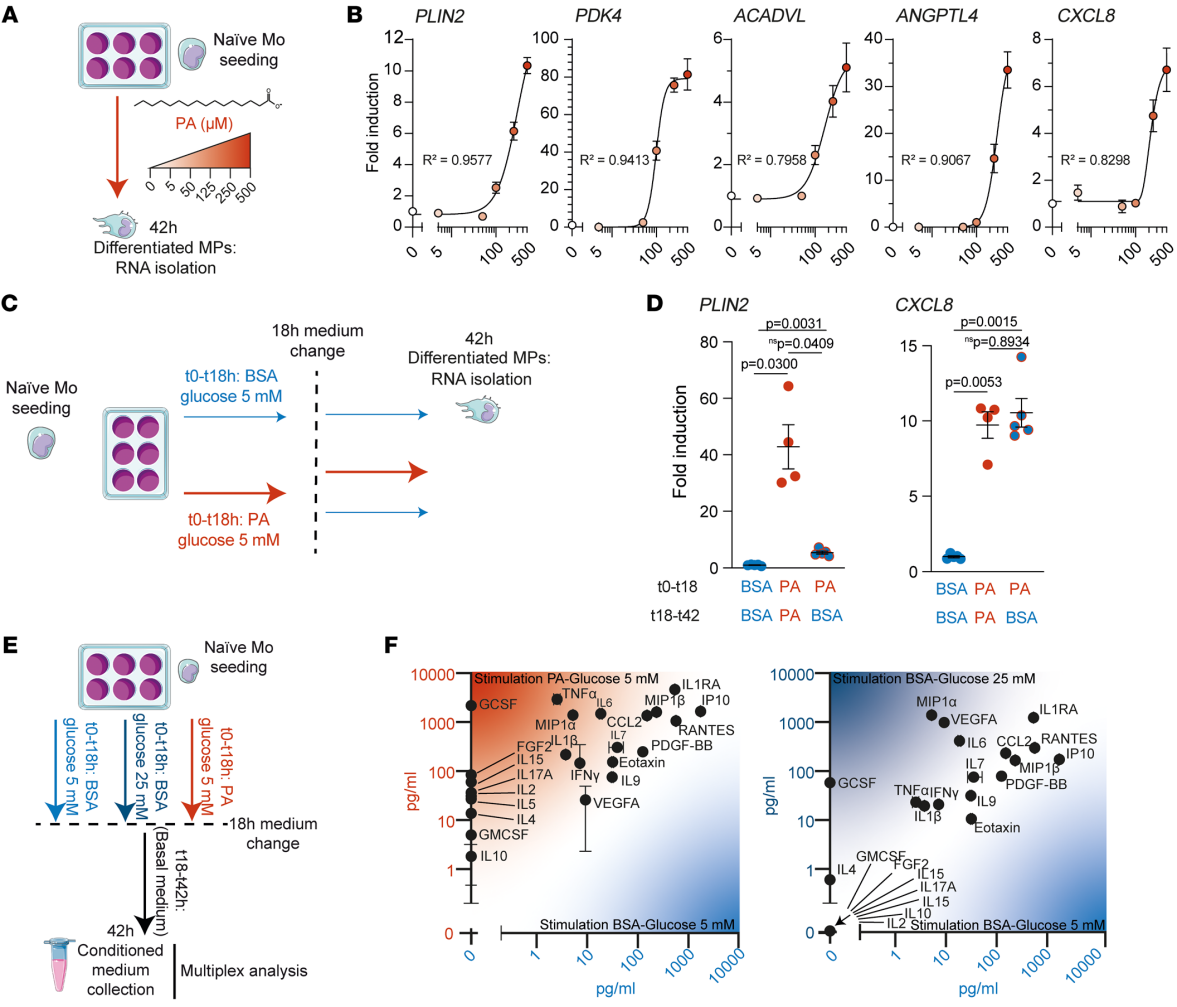
Lipid-associated MPs produce inflammatory cytokines. (**A**) Schematic representation of naive Mo treatment with increasing concentrations of PA. (**B**) Nonlinear regression representation of the RT-qPCR relative expression of selected markers of healthy donor naive Mos treated for 42 hours with increasing concentrations of PA (0–500 μM). Values represent the mean ± SEM of 5 independent culture points. Goodness of fit is indicated by *R^2^* (0.9577, 0.9413, 0.7958, 0.9067, and 0.8298, respectively). (**C**) Schematic representation of 2-phase treatment of naive Mos to test the long-term effect of PA. (**D**) Scatter plot representation of RT-qPCR expression of *PLIN2* and *CXCL8* in healthy donor naive Mos subjected to the 2-phase treatment. Values represent the mean ± SEM of a minimum of 4 independent culture points. *P* values were determined by 1-way Welch’s ANOVA (*P* = 0.0010 for *PLIN2*; *P* = 0.0002 for *CXCL8*) followed by Dunnett’s T3 multiple-comparison test. (**E**) Schematic representation of the preparation CM of Mos. (**F**) Scatter plot of cytokines and growth factor protein concentrations in pg/mL detected by multiplex measurements in CM of naive Mos from a healthy donor differentiated with PA (PA-bound BSA, *y* axis) or BSA (unbound BSA, *x* axis) (left graph) or 25 mM glucose plus BSA (*y* axis) or 5 mM glucose plus BSA (*x* axis) (right graph).

We next explored naive Mo differentiation by assessing the effects of different concentrations and different durations of PA stimulation. Concentrations above 100 μM PA induced dose-dependent expression of *PLIN2*, *PDK4*, *ACADVL*, *ANGPTL4*, and *CXCL8* transcripts after 42 hours (Figure 4, A and B). We next compared the expression of *PLIN2* and *CXCL8*, which we demonstrated to be markers of lipid overload in early differentiating Mos, after 42 hours by Mos stimulated with PA for only the first 18 hours and compared the expression levels with expression by Mos continuously stimulated for 42 hours (Figure 4C). Medium renewal after 18 hours (PA-to-BSA condition) resulted in a reduction of *PLIN2* expression compared with the PA-to-PA condition (PA exposure from *t*0 to *t*42 h), but *CXCL8* expression remained elevated (Figure 4D). Our results demonstrate that initial PA stimulation may be sufficient to induce a sustained inflammatory response.

### Lipid-stimulated MPs secrete inflammatory cytokines.

The presence of lipid-laden MPs in the vicinity of vascular leaks could be a source of vasoactive cytokines. We thus collected conditioned medium (CM) 42 hours after an initial 18-hour stimulation of naive Mos with PA or 25 mM glucose (Figure 4E). Using multiplex technology, the cytokine content of PA-stimulated PA-free CM (PAstimCM^PAfree^) (PA-to-BSA) was compared with CM from MPs previously stimulated with control CM (CtlCM) (BSA-to-BSA) or with the CM from MPs previously stimulated with 25 mM glucose (Figure 4E). Early PA stimulation upregulated the expression of the cytokines CCL2, FGF2, GMCSF, IL-1β, IL-2, IL-4, IL-5, IL-6, IL-10, IFN-γ, and TNF-α, which have been found to be elevated in the vitreous of patients with DR (5). By contrast, these cytokines were only slightly modulated or not expressed after a similar early stimulation with 25 mM glucose (Figure 4F).

These results highlight that key DR inflammatory cytokines were upregulated in the secretome of MPs after an initial stimulation with PA. We have previously showed that PA_CH3_, the unreadily metabolizable analog of PA, reduces lipid polarization of MPs (Figure 2E and Supplemental Figure 2B). We therefore hypothesized that PA_CH3_ could reduce the expression of a number of DR-related cytokines compared with PA. We thus examined the expression of *CXCL8*, *TNF*, *IL1B*, and *IL6* and found that all of these inflammatory cytokine transcripts were significantly decreased when naive Mos were exposed to PA_CH3_ instead of PA (Supplemental Figure 4A).

### Lipid-associated MPs show vasodegenerative properties.

**Figure 5:**
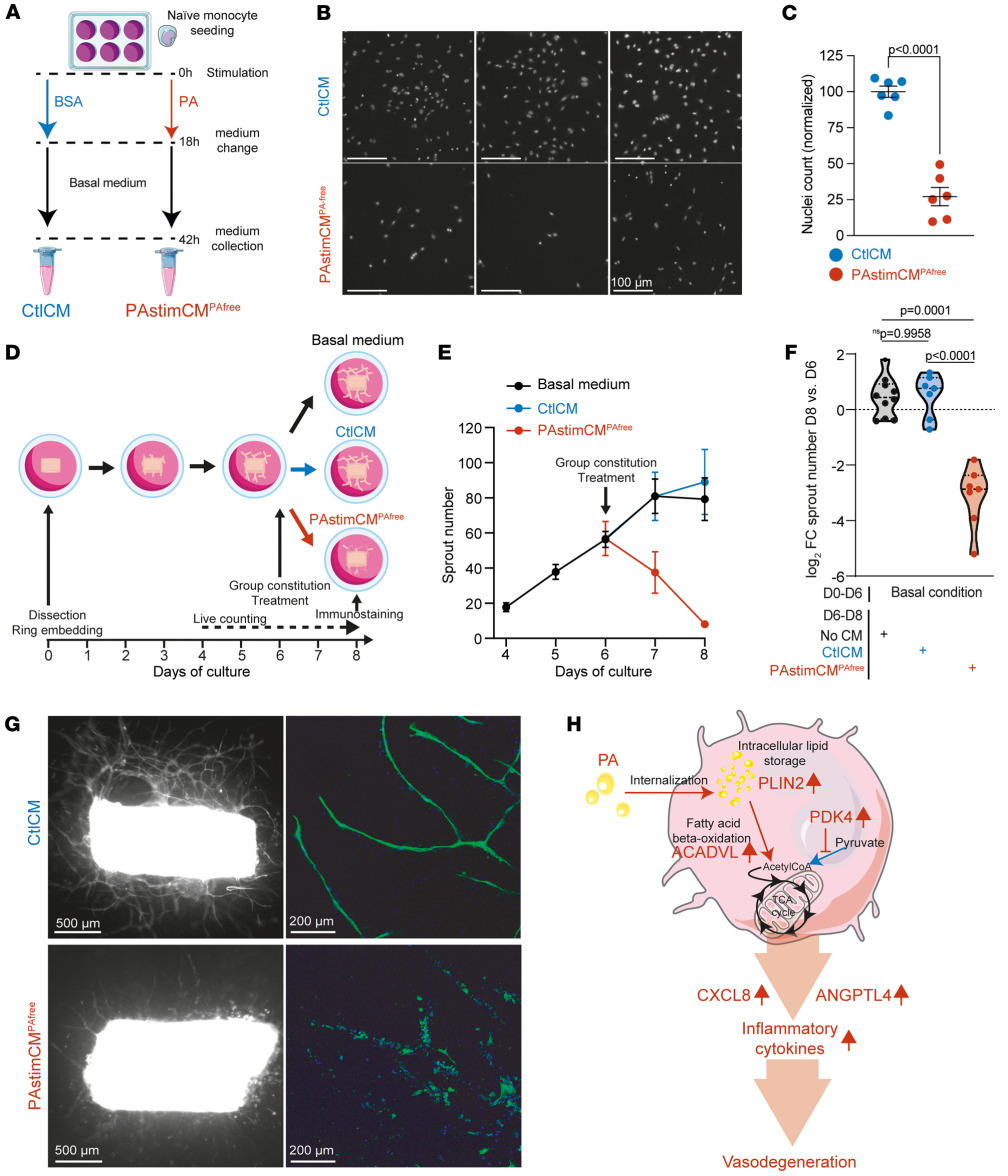
Lipid-associated MPs show vasodegenerative properties. (**A**) Schematic representation of the preparation of CtlCM and PAstimCM^PAfree^ from healthy donor Mos treated with PA (PA-bound BSA) or BSA (unbound BSA). (**B** and **C**) HUVECs were stimulated with either CtlCM or PAstimCM^PAfree^. (**B**) Epifluorescence images of HUVEC nuclei stained with Hoescht (white). Scale bars: 100 μm. (**C**) Scatter plot of normalized nuclei counts. Values represent the mean ± SEM of 6 independent culture points. The *P* value was determined using Welch’s 2-tailed *t* test. (**D**–**G**) Capillary degeneration was quantified in an ex vivo assay (48). (**E**) Time-course of the mean ± SEM sprout number between day 4 (D4) and day 8 (D8) in the 3 treatment groups: basal medium (*n* = 10, black), CtlCM (*n* = 7, blue), and PAstimCM^PAfree^ (*n* = 7, red). (**F**) Violin plot of the log_2_ FC of sprout numbers between paired day-6 and day-8 rings; dots represent individual aortic rings, and dashed lines represent the median and quartiles. *P* values were determined by 1-way Welch’s ANOVA test (*P* < 0.0001) followed by Dunnett’s T3 multiple-comparison test. (**G**) Aortic rings and sprouts treated with CtlCM or PAstimCM^PAfree^ on day 6 and stained with COL4 on day 8. Left: Epifluorescence micrographs of COL4 (white). Scale bars: 500 μm. Right: Higher-magnification confocal micrographs of COL4 (green). Scale bars: 200 μm. Nuclei were stained with Hoechst (blue). (**H**) Schematic of the biological changes in Mos after lipid exposure and their acquired properties.

We tested the activity of the CM on human umbilical vein endothelial cells (HUVECs). PAstimCM^PAfree^ resulted in a 4-fold reduction in cell numbers after 24 hours compared with CtlCM (Figure 5, A–C). We next quantified the capillary degeneration induced by PAstimCM^PAfree^ using our specially designed ex vivo assay (48). Rat aortic rings were allowed to sprout for 6 days and were then treated with PAstimCM^PAfree^, Ctl-CM, or basal medium. Sprouts from all rings were counted daily from day 4 (2 days before treatment) to day 8 (2 days after treatment) (Figure 5D). CtlCM did not affect the sprouting rate of aortic rings. On the contrary, the addition of PAstimCM^PAfree^ resulted in a decrease in the mean number of branches starting from day 7 (Figure 5E), and a paired analysis of the numbers of branches between day 6 and day 8 showed severe loss of the organized endothelial cell network in each individual aortic ring (Figure 5F). Lectin-stained images of day-8 fixed aortic rings confirmed capillary degeneration after the addition of PAstimCM^PAfree^ (Figure 5G). Stimulation with PA_CH3_ did not correlate with increased expression of inflammatory cytokines (Supplemental Figure 4A). Conversely, PA_CH3_stim-CM^PACH3free^ (where naive Mos were initially stimulated with PA_CH3_ instead of PA) demonstrated significantly lower vasodegenerative activity compared with PAstimCM^PAfree^ (Supplemental Figure 4, B and C). Given the design of our study, we concentrated only stable macromolecules larger than 10 kDa that were present in the CM. Consequently, our results suggest the likely involvement of macromolecules such as inflammatory cytokines secreted by MPs in the observed vasodegenerative properties (Figure 5H).

### Inhibiting PPARγ signaling normalizes the PA-induced phenotype of differentiated MPs.

**Figure 6:**
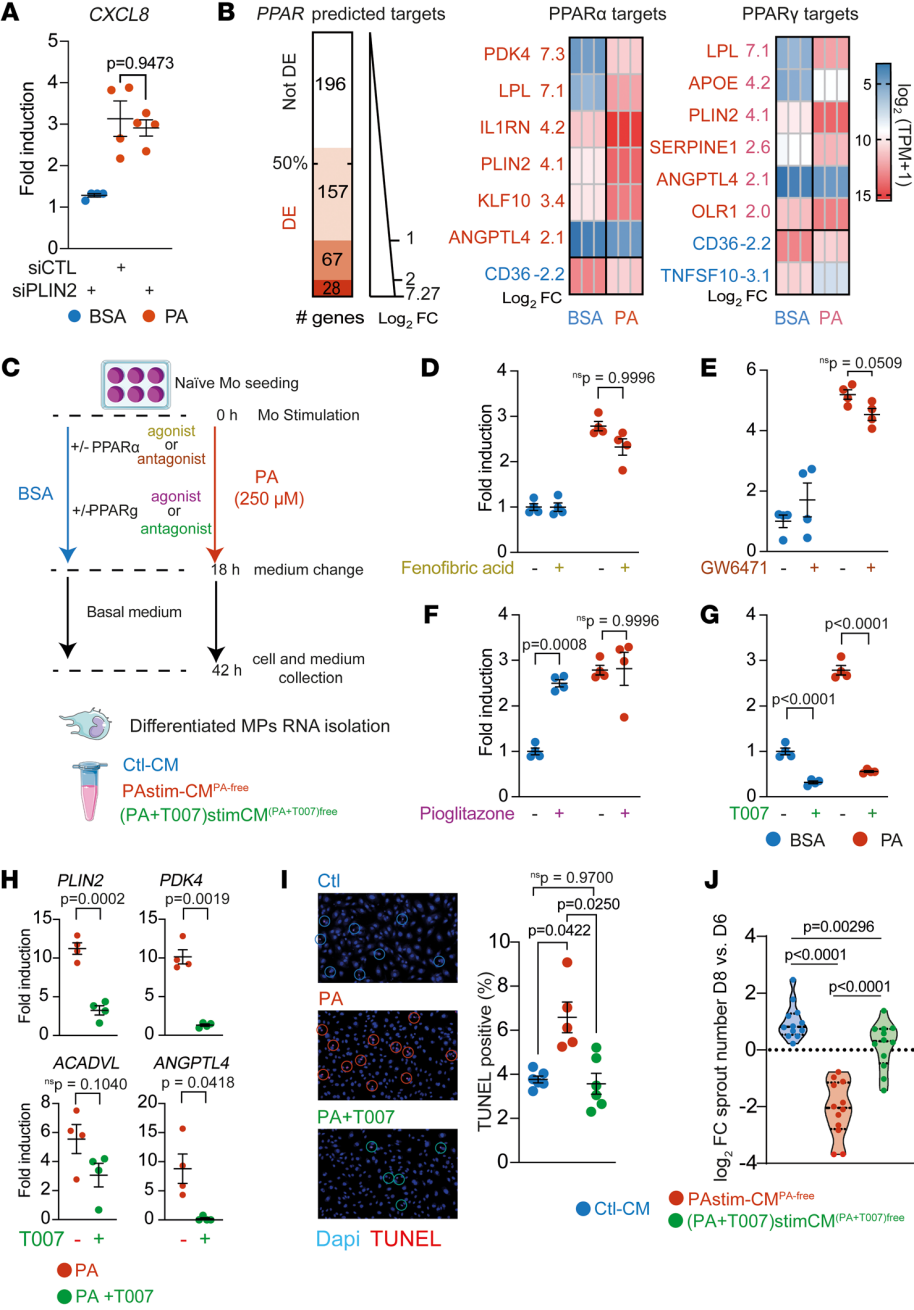
Inhibition of PPARγ signaling normalizes the PA-induced lipid-associated phenotype. (**A**) Scatter plot of RT-qPCR quantification of *CXCL8* in THP-1 cells transfected with the indicated siRNA and treated with BSA (unbound BSA) or PA (PA-bound BSA). *P* values were determined by 1-way Welch’s ANOVA test (*P* = 0.0136) followed by Dunnett’s T3 multiple-comparison test. (**B**) Left: Graph of high-confidence predicted PPAR target genes (49) differentially expressed in naive Mos treated with PA. Middle and right: Heatmaps of the expression level (TPM) of curated PPARα target genes with a log_2_ FC of 2 or higher (middle) and curated PPARγ target genes with a log_2_ FC of 2 or higher (right). (**C**) Schematic of the stimulation of naive Mos with PA or BSA and PPAR modulators. (**D**–**G**) Scatter plots of RT-qPCR quantification of *CXCL8* in healthy donor naive Mos treated with either PA or BSA and with (**D**) fenofibric acid PPARα agonist (*P* values for interaction, *P* = 0.0790; PA effect, *P* < 0.0001; and drug effect, *P* = 0.0760); (**E**) GW6471 PPARα antagonist (*P* = 0.0570, *P* < 0.0001, and *P* = 0.9448); (**F**) pioglitazone PPARγ agonist (*P* = 0.0027, *P* = 0.0002, and *P* = 0.0021); or (**G**) T0070907 (T007) PPARγ antagonist (all *P* < 0.0001). Interactions and *P* values and were determined by 2-way ANOVA followed by Tukey’s multiple-comparison test. (**H**) Scatter plots of RT-qPCR quantification of the indicated genes in healthy donor naive Mos treated with PA plus T007. *P* values were determined using a *t* test with Welch’s correction. (**I**) Representative images and scatter plot of TUNEL^+^ HUVECs after stimulation with CtlCM, PAstimCM^PAfree^, or PAstim+T007-CM^PA&T007-free^. *P* values were determined by 2-way Welch’s ANOVA (*P* < 0.0046) followed by Dunnett’s T3 multiple-comparison test. (**J**) Violin plot of the log_2_ FC of sprout numbers between paired rings before and after stimulation with CtlCM, PAstimCM^PAfree^, or PAstim+T007-CM^PA&T007free^. Dashed lines indicate the median and quartiles. *P* values were determined by 1-way Welch’s ANOVA (*P* < 0.0001) followed by Dunnett’s T3 multiple-comparison test.

We found a strong association between *PLIN2* and *CXCL8* expression in the presence of T2DM plasma or PA. However, we show that lipid removal led to a reduction in *PLIN2* expression, whereas *CXCL8* overexpression remained persistent, suggesting that the regulation of *PLIN2* and *CXCL8* may be uncoupled after the initial period of lipid exposure (Figure 4D). We therefore next aimed to determine whether an initial *PLIN2* upregulation is necessary for *CXCL8* induction. Given the short lifespan of primary human Mos and the challenges associated with their transfection for siRNA assays, we used as a model the more easily transfectable THP-1 cells, a human monocytic cell line derived from a patient with acute monocytic leukemia. The response of THP-1 cells transfected with a control siRNA to PA was similar to that of primary human Mos with a strong upregulation of *PLIN2* and *CXCL8* (Supplemental Figure 5, A and B). siRNA targeting *PLIN2* reduced *PLIN2* expression by 77% (Supplemental Figure 5A) and reduced PLIN2 induction in THP-1 cells by 69 % in the presence of PA (Supplemental Figure 5B). This reduced expression of *PLIN2* did not reduce inflammatory *CXCL8* expression (Figure 6A) or lipid metabolism markers in THP-1 cells (Supplemental Figure 5C).

Therefore, we considered a broader approach targeting signaling pathways associated with lipid exposure. Our comprehensive bulk RNA-Seq analysis revealed that naive, healthy MPs swiftly responded to PA exposure by upregulating the expression of *PPARG*, a gene encoding a member of the PPAR transcription factor family. Conversely, the expression of *PPARA*, which encodes the therapeutic target of fenofibrate — a clinical diabetes treatment — was marginally downregulated (log_2_ FC = –0.44). Given the critical role of PPARs in controlling the transcription of lipid metabolism–associated genes, we hypothesized that a potentially large-scale, PPAR-mediated modulation of lipid-associated phenotypic characteristics occurs in MPs. Consequently, we examined the expression of PPARα and PPARγ transcriptional targets in PA-stimulated Mos. We assessed the expression of 448 high-confidence PPAR target genes that were identified by Fang et al. using a combination of transcriptomics data and the presence of the PPAR-responsive element (PPRE) in the regulatory regions of these target genes (49). We found that 252 of them (56.2%) were statistically differentially expressed after PA exposure (Figure 6B and Supplemental Table 6). Next, we analyzed the regulation of the 83 manually curated transcript targets of PPARα and the 104 manually curated transcript targets of PPARγ (49). PA regulated 38 transcripts (45.7%) on the curated PPARα target list, including 7 (8.4%) with a log_2_ FC of 2 or higher (Figure 6B and Supplemental Table 7). PA also regulated 50 transcripts (49.0%) on the curated PPARγ list, including 6 (5.8%) with a log_2_ FC of 2 or higher (Figure 6B and Supplemental Table 8). Although *ACADVL* was not included in the curated PPAR target list, the other 3 previously identified PA-associated transcripts (*PLIN2*, *PDK4*, and *ANGPTL4*) were all represented in the highly regulated target list (Figures 2 and 3). These findings suggest that PPARα and PPARγ are potential targets to blunt lipid-induced proinflammatory MP polarization.

We therefore tested the effects of fenofibric acid and GW6471 (a PPARα agonist and antagonist, respectively) and pioglitazone and T0070907 (a PPARγ agonist and antagonist, respectively) on the expression of the selected prototypical *CXCL8* transcript (Figure 6, C–G). Neither the PPARα agonist nor the antagonist affected *CXCL8* expression in the BSA condition, and these agents failed to reduce its induction after PA stimulation (Figure 6, D and E). Pioglitazone, a PPARγ agonist, increased *CXCL8* expression in the BSA condition and had no effect after PA stimulation (Figure 6F). Finally, the PPARγ antagonist T0070907 strongly reduced *CXCL8* expression after PA stimulation and also reduced its basal expression in the BSA condition (Figure 6G). Consistent with these results, T0070907 reduced the expression of the PPAR targets *PLIN2, PDK4*, and *ANGPTL4* but not of the non-PPAR–curated target *ACADVL* (Figure 6H). *CXCL8* downregulation was associated with decreased expression of the DR-associated cytokines *TNF*, *IL1B*, and *IL6* (Supplemental Figure 6).

We have previously shown that PAstimCM^PAfree^ leads to a reduction in the number of HUVECs relative to CtlCM (Figure 5, A–C). To gain further knowledge about the potential protective effect of T0070907 on endothelial cell viability, we next analyzed HUVEC apoptotic cell death by TUNEL assay 48 hours after addition of CtlCM, PAstimCM^PAfree^, or (PA+T0070907)stimCM^(PA+T0070907)free^. PAstimCM^PAfree^ induced a statistically significant 75% increase in TUNEL^+^ HUVECs compared with CtlCM, whereas (PA+T0070907)stimCM^(PA+T0070907)free^ completely rescued HUVECs undergoing apoptotic cell death (Figure 6I). Finally, we compared the effect of these 3 CMs on the integrity of the capillary network in the rat aortic ring model. Again, we found that (PA+T0070907)stimCM^(PA+T0070907)free^ was protective of the vascular network compared with PAstimCM^PAfree^ (Figure 6J).

## Discussion

In this study, we show that PLIN2^+^ MPs were present in the vicinity of leaky capillaries, identified by extraluminal ALB, in postmortem human T2DM donor retina with histopathologic features of NPDR. Importantly, we demonstrate that hi-plasma from patients with T2DM (with or without DR) strongly upregulated the expression of lipid overload markers in Mos from healthy donors, which strongly correlated with the expression of DR biomarkers such as *ANGPTL4* and *CXCL8*. We also show that PA, an abundant plasma FFA that is elevated in dyslipidemic plasma of patients with T2DM, induced a nearly identical expression pattern in naive human Mos. We characterized the PA-induced transcriptome and secretome changes and show that the PA-stimulated Mo secretome induced endothelial apoptosis and ex vivo capillary degeneration. A PPARγ antagonist blunted PA-induced cytokine expression and reversed the vasodegenerative properties of the PA-stimulated Mo secretome. This study provides comprehensive insights into the glycemia-independent activation of Mos in T2DM and identifies MP PPARγ as a target for dyslipidemia-activated MPs in DR.

### Plasma lipids are internalized by Mos.

FFAs are abundant in the blood, and their levels are elevated in T2DM plasma (34, 50). TGs, another lipid component found in the blood, are composed of 3 FAs that can be hydrolyzed by extracellular LPL and further increase the pool of FFAs available to cells. Lipoprotein TGs and ALB-bound FFAs do not extravasate in the absence of vascular leakage. However, years of diabetes can compromise the integrity of the blood-retina barrier, leading to increased permeation of plasma components such as ALB, carbohydrates, and lipids into the retinal environment. These elements not only come into direct physical contact with local cells such as Müller glial cells, leading to their robust activation (51, 52), but also shape the milieu that drives the differentiation of infiltrating Mos. Using chromatographic techniques, we demonstrated that Mos internalized PA. We show that the proportion of PA chains increased markedly, while the proportion of other FA chains did not change. Together with the increased expression of PLIN2, this suggests that PA was stored intracellularly. Interestingly, we show that Mo activation persisted even after PA removal. This long-term effect suggests that the resolution of in vivo inflammation may lag even after extravasated lipids have been taken up intracellularly.

Clinically, elevated plasma TG levels have been associated with an increased risk of DR, with a clear association between TG levels and an increased frequency of hard exudates (53). We performed classical lipid analysis of plasma from our donors in the clinic (total cholesterol [TC], HDL cholesterol [HDLc], LDLc, VLDLc, and TGs) and observed no significant difference in overall plasma lipid load between the controls and T2DM donors in our cohort. However, we found that plasma-derived nonglucose cues could induce lipid-associated MP polarization. Our study thus suggests that there may be undiagnosed risks (i.e., the available PA that we showed had a detrimental effect on Mo polarization) of Mo activation that cannot be detected by performing classical lipid analysis in patients with T2DM. Our study focused on PA, the most abundant FFA in the blood. PA is not the only elevated FFA in diabetes, and numerous FFAs are also found to be elevated in T2DM (34, 35). We also show that SA, the second most abundant saturated FFA in the blood, PoA, the n-7 unsaturated form of PA, and a plasma representative lipid blend also induced *PLIN2* overexpression.

### Lipid-laden MPs are associated with vascular remodeling during DR.

PLIN2^+^ MPs are found in atherosclerosis plaques, cholesterol polyps of the gall bladder, and fat necrosis (37). In the present study, in DR, we found PLIN2^+^ MPs in regions of microvascular disease characterized by ALB extravasation, suggesting that MPs were involved in the uptake of extravasated lipids and lipid storage. A similar observation has been made in the central nervous system, where PLIN2^+^ cells accumulate with age in the hippocampus and can be stained with lipid dyes (38). Consistent with our observation, MPs have been found in the red oil^+^ hard exudates of the retina in DR (54). More recently, adaptive optics funduscopy has revealed the presence of round motile particles that resemble MPs in hard exudates, a structure found in the periphery of regions of active vascular leakage (43). Collectively, these findings indicate that lipid-polarized MPs are found in regions of active vascular remodeling in the DR retina, that they may progressively accumulate in regions exposed to plasma leakage, and that they could be a source of vasoactive cytokines.

In an effort to understand how plasma leakage may influence MP activation, in particular the fate of infiltrating Mos, we differentiated naive Mos in the presence of hi-plasma from healthy and T2DM donors. We show that hi-plasma from patients with T2DM rapidly induced a lipid-associated polarization of MPs characterized by overexpression of lipid-related genes. We also show a correlation between *PLIN2* expression (used as a proxy for MP lipid load) and other lipid-related genes as well as DR biomarkers such as *ANGPTL4* and *CXCL8*.

Such differentiation of Mos could be mimicked by PA, but not by glucose exposure. PA stimulation was associated with an increase in the production of at least 14 proinflammatory cytokines, 11 of which have been shown to be directly involved in DR. Similarly, Müller glial cells, the major glial cells of the retina, have been shown to have a greater increase in the expression of cytokines associated with DR when stimulated with PA than with glucose (51, 52).

ANGPTL4 is a protein that regulates lipid processing but also has vasoactive properties. ANGPTL4 was notably upregulated when naive Mos were stimulated with PA or T2DM plasma, and *ANGPTL4* expression showed a strong correlation with *PLIN2* expression. ANGPTL4 plays an important role in the pathogenesis of DR (55, 56). Its expression has been found to correlate with the nonperfused area (45); in line with this finding, we found that the net effect of the PA-stimulated MP secretome was vasodegenerative, which was highlighted by a decrease in vascular sprouting in a 3D model and an increase in endothelial cell apoptosis.

Interestingly, we found that MPs remained activated and secreted inflammatory cytokines for at least 24 hours after the initial lipid stimulation. This effect may lead to a long-term vascular remodeling activity of lipid-stimulated MPs. Such vasodegenerative activity is consistent with the results of previous reports showing an overall antiangiogenic activity of MPs in the context of retinal ischemia. Indeed, MPs are associated with early vascular regression in models of ischemic retinopathy and contribute to the resolution of vascular proliferation (10, 57). In our hands, mouse Mos were less sensitive to PA than were human Mos, and our attempts to establish a mouse model of lipid-associated DR have so far been unsuccessful. This difference is consistent with the overall difference in the severity of DR between mice and humans. Mice do not develop clear early signs of NPDR, such as severe retinal capillary loss or massive blood retina-barrier breakdown and thus are not prone to progress to PDR like humans are. Hence, although our ex vivo aortic ring model clearly identified vasodegenerative properties of lipid-exposed MPs, this could not yet be validated in vivo. In contrast, subretinal MPs in exudative age-related macular degeneration show proangiogenic activity (58, 59). The exact mechanism by which the addition of CM from PA-stimulated MPs leads to endothelial cell loss is yet to be determined. Cytokines found to be increased in CM from PA-stimulated MPs (CCL2, FGF2, IL-1β, IL-2, IL-4, IL-5, IL-6, CXCL8, IL-10, IFN-γ, and TNF-α) have been implicated in the pathogenesis of DR and are probably responsible for this activity.

### The PPARγ signaling pathway is involved in vasodegenerative activities of lipid-associated MPs.

We demonstrated a potent correlation between the expression of *PLIN2* and *CXCL8* during the early stages of MP differentiation in the context of plasma lipids and exposure to various isolated FFAs. This may suggest a regulatory role for PLIN2 in *CXCL8* expression. Similar correlations between PLIN2 and inflammatory cytokines have been observed in tumor-associated macrophages as well as in THP-1 cells (39, 60). Notably, PLIN2^+^ MPs are observed around retinal microaneurysms and in numerous pathological contexts, suggesting that therapeutic inhibition of PLIN2 may serve as a strategy to curb inflammation-associated vascular remodeling. However, our results show that siRNA-based silencing of *PLIN2* transcript expression in the THP-1 cell line, a model of Mos, neither inhibited *CXCL8* transcription nor altered the regulation of other selected markers of lipid metabolism. Similarly, the production of cytokines by bone marrow–derived macrophages stimulated with various lipids remained unaltered following *PLIN2* deletion (61), whereas Chen et al. reported a slight reduction in inflammatory cytokine expression in unstimulated THP-1 cells following *PLIN2* silencing (39). Altogether, these results suggest that, while PLIN2 serves as an excellent marker of lipid polarization in vivo in the human retina and in vitro with Mos stimulated by T2DM plasma or PA, its inhibition by siRNAs is insufficient to prevent inflammatory polarization of at least lipid-exposed THP-1. Considering that we observed persistent overexpression of *CXCL8*, even after PA removal, and given the high stability of PLIN proteins when incorporated into lipid droplets (62, 63), therapeutic strategies targeting the inhibition of PLIN2 protein production may prove ineffective in situations in which MPs harboring lipid droplets have already accumulated.

Our transcriptomics analysis of differentiating Mos in the presence of PA or plasma from patients with T2DM showed upregulated expression of the genes *PLIN2*, *PDK4*, and *ANGPTL4*. These genes are all transcriptional targets of PPAR transcription factors. Consistent with this role, our RNA-Seq analysis identified *PPARG* as one of the most highly upregulated genes (log_2_ FC = 4.07), whereas *PPARA* was slightly downregulated (log_2_ FC = –0.44). Members of the PPAR family are nuclear transcription factors that bind to the retinoid X receptor α (RXRα) to regulate the expression of numerous genes, most of which are part of the lipid metabolism pathway. PPARs act as repressors when not bound to a ligand. When natural (i.e., FFA agonists) or synthetic ligands bind to the ligand-binding domain (LBD) of PPARs, they promote the binding of coactivator or corepressor factors, resulting in ligand-dependent modulation of gene expression. We investigated the role of the PPARα and PPARγ pathways in the differentiation of Mos in the presence of lipids using PPARα and PPARγ agonists and antagonists. Fenofibrate, which is metabolized to the PPARα agonist fenofibric acid, is a widely used systemic treatment to lower plasma lipid concentrations. Fenofibrate has successfully proven its protective effect in patients with DR (27, 28). It has also been shown to reduce systemic inflammation in patients with NPDR, suggesting a potential role of circulating lipids or lipid metabolism in the chronic inflammatory status of patients with T2DM (64). Fenofibric acid and GW6471, a PPARα antagonist, did not regulate the expression of the DR prototypical cytokine *CXCL8* in MPs. However, in animal models of T1DM, systemic fenofibrate treatment has a protective effect on the blood-retina barrier and protects against neovascularization in the oxygen-induced retinopathy model, notably by attenuating the overexpression of VEGF, CCL2, and ICAM-1 (39). Overall, this suggests that the beneficial effects of PPARα agonists in vivo could be due to effects on nonmyeloid cells. For example, PPARα agonists have been shown to directly protect endothelial cells (65, 66).

In contrast to the PPARα agonist T0070907, a PPARγ antagonist, attenuated PA-induced *CXCL8* overexpression and the CM of naive Mos treated with PA+T0070907 showed reduced vasodegenerative properties. Activation of the PPARγ pathway in MPs is generally considered to be antiinflammatory (67, 68), and there is consensus for a beneficial effect of systemic PPARγ agonist treatment in a wide range of diseases, including diabetes and its comorbidities. However, PPARγ antagonism has been reported to protect against the progression of nonalcoholic fatty liver disease (NAFLD) (69) and to reduce foam cell formation (70–72). In BV2 microglia cells treated with LPS, and thus in the absence of DR-relevant stress, *Pparg* knockdown or T0070907 treatment was also shown to be associated with antiinflammatory polarization (73). In a study using the human myeloid cell line Mono Mac 6, the small molecule bindarit was shown to promote, in a PPARγ-dependent manner, overexpression of the lipid-associated protein FABP4 along with the *CXCL8* transcript (74). In accordance with this result, we found that pioglitazone, a PPARγ agonist, increased the expression of *CXCL8* in the BSA-only condition, suggesting that PPARγ upregulation may result in adverse effects even in the absence of exogenously added PPAR ligand. Locally in the eye, inflammation in retinal pigment epithelial cells treated with *N*-retinylidene-*N*-retinylethanolamine, a byproduct of the visual cycle associated with age-related macular degeneration, was also shown to be inhibited by T0070907 (75). A possible explanation for this discrepancy between the beneficial effects of PPARγ agonists and antagonists may be that normalization of circulating lipid levels and restoration of lipid storage in adipocytes by systemic PPARγ agonism is responsible for the observed beneficial effect, whereas in our setting, PPARγ antagonism was applied only to MPs. Overall, growing evidence suggests that the definition of PPARγ pathway activation as an inhibitor of M1 inflammatory polarization and an inducer of M2 antiinflammatory polarization may be an oversimplification. The mechanism by which these plasma lipids induce Mo differentiation into MPs with proinflammatory properties is still unknown. However, and in accordance with our finding that PA was rapidly internalized, PA-induced polarization of MPs to a proinflammatory profile has recently been shown to be independent of direct TLR4 binding (76). Our results further suggest that the differentiation of Mos in the presence of lipids leads to a modification of their metabolism, as (a) the use of PA_CH3_, a PA analog that can be internalized but that is not readily metabolizable, did not trigger CXCL8 secretion, nor did it induce the degeneration of aortic ring sprouts, and (b) the *PDK4* gene was found to be highly upregulated in the presence of PA, suggesting a potential switch to aerobic glycolysis (the so-called Warburg effect), a situation found in proinflammatory MPs. Of note, PPARδ antagonism is also beneficial in the ocular context. The lipid-induced Müller glial cell response can be inhibited by GSK0660, a PPARδ antagonist (77). Overall, this suggests that PPAR antagonists could be used locally in addition to systemic PPARα agonist treatment to inhibit the pathological differentiation of local and infiltrating cells in the retina in DR.

### Study limitations.

In this study, we focused on identifying the major class of lipids present in the blood of our cohort of patients. However, we must acknowledge that detailed information regarding lipid chain composition and concentrations of specific FFAs, including PA, was not obtained. Future investigations will be necessary to establish possible correlations between individual plasma lipid species, the activation level of naive Mos, and DR and its progression.

Additionally, our findings demonstrated that the secretome of PA-stimulated MP exhibits antiangiogenic activity, suggesting a potential link between lipid extravasation and vascular degeneration in vivo. However, it is essential to recognize that our study lacked relevant in vivo models of vascular permeability. As a result, we were unable to assess the relative importance of MP-induced degeneration compared with other known circulating factors involved in vascular remodeling, such as high glucose, advanced glycation endproducts (AGEs), oxidized lipids, and metabolites. More recently, diabetes-induced microbiote modification has also been recognized as an important player in disease progression. Exploring additional in vivo models and investigating the interactions of various factors contributing to vascular remodeling will provide valuable insights into this complex phenomenon.

In conclusion, DR is a neurodegenerative, microvascular, and inflammatory complication of T2DM, in which breakdown of the blood-retina barrier and vascular degeneration lead to the extravasation of plasma proteins, lipids, and leukocytes. We showed that MPs expressing PLIN2 were found in retinas with active vascular leakage and vascular remodeling. Exposure of naive Mos to hi-plasma from patients with diabetes or PA was sufficient to induce *PLIN2* expression and trigger long-term expression of DR-related proinflammatory cytokines. The secretome of lipid-exposed naive Mos showed strong vasodegenerative properties that could be blunted by a PPARγ inhibitor. Our study sheds light on the harmful role of lipid-associated MP accumulation in the retina in DR and the role of pathogenic lipids, rather than glucose, in the activation of these cells.

## Methods

Detailed Methods can be found in the Supplemental Methods.

### FFA solubilization and medium preparation.

To allow FFA solubilization in culture medium, FFAs were bound to FFA-free BSA. FFAs were dissolved in absolute ethanol (EtOH) and added to a BSA-containing culture medium (0.88% w/v of BSA) to obtain the working concentration (500 μM FFA, 0.5 % v/v EtOH; FFA/BSA molar ratio of 3.8).

### Preparation of CM.

To obtain CM free of BSA and PA, Mos were isolated as described in the Supplemental Methods and stimulated for the first 18 hours. The culture medium was then removed, and early differentiated MPs were cultured for another 24 hours in a fresh control culture medium.

### Statistics.

GraphPad Prism 8 (GraphPad Software) was used for all graphical representations and for all but the RNA-Seq statistical analysis. A detailed explanation of the specific statistical choices made is provided in the Supplemental Methods. A *P* value of less than 0.05 was considered significant.

### Study approval.

Plasma samples from patients with T2DM and healthy donors were collected from volunteer donors at the Institute of Ophthalmology Foundation Conde de Valenciana (Mexico City, Mexico), in accordance with Declaration of Helsinki principles and with the approval of the Investigation Committee (registry 13 CI 09 015 261, protocol number CI-051-2015); the Research Ethics Committee (registry 13 CEI 09 015 095, protocol number CEI-2015-11-05); and the Biosafety Committee (registry 13 CB 31 050 269, protocol number CB-051-11-2015) (all in Mexico City, Mexico). Individuals provided written informed consent prior to their participation in the study.

### Data and materials availability.

Experimental materials are available upon request with no restrictions. The RNA-Seq raw fastq files and count files were deposited in the NCBI’s Gene Expression Omnibus (GEO) database (GEO GSE239512). Values for all data points in graphs can be found in the Supplemental Supporting Data Values file.

## Author contributions

GB, RK, FB, and XG conceived the study. GB, HCM, FB, AJC, and XG curated data. GB, HCM, FS, and XG acquired funding. GB, RK, LP, TMS, HCM, PN, SA, FPMR, LV, and XG collected data. GB, RK, and XG designed the study methodology. AC, DRDLP, AJC, YG (patient recruitment and phenotyping), NA (lipidomics), and JAS provided resources. GB, NA, YG, FS, CD, and XG supervised the study. GB and XG wrote the original draft of the manuscript. GB, RK, HCM, RK, FPMR, DRDLP, AJC, CD, YG, FS, and XG reviewed and edited the manuscript.

## Supplementary Material

Supplemental data

Supplemental table 1

Supplemental table 10

Supplemental table 2

Supplemental table 3

Supplemental table 4

Supplemental table 5

Supplemental table 6

Supplemental table 7

Supplemental table 8

Supplemental table 9

Supporting data values

## Figures and Tables

**Table 1:**
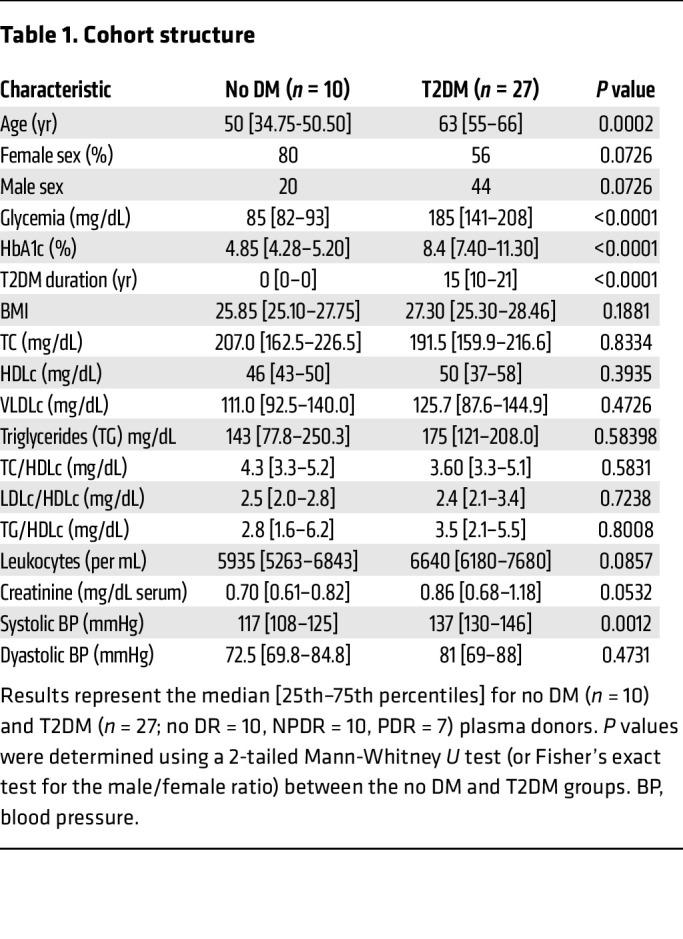
Cohort structure

## Version 1 10/02/2023

Electronic publication
