# Supplementary material for: Perilipin 2–positive mononuclear phagocytes accumulate in the diabetic retina and promote PPARγ-dependent vasodegeneration
Source: J Clin Invest. 2023 Oct 2;133(19):e161348. doi: 10.1172/JCI161348 (PMC10702478; doi:10.1172/JCI161348)
Supplement: Supplemental data [file jci-133-161348-s255.pdf]

Supplementary figures

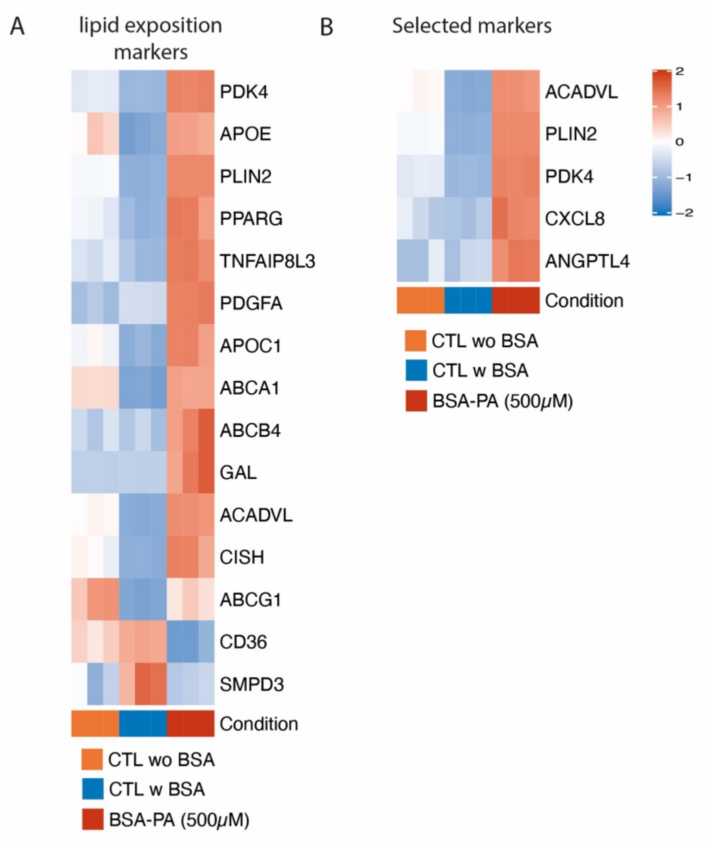

Supplemental Figure 1. Comparison of lipid exposition markers and DR associated gene in the absence or presence of BSA. RNAseq transcriptomic analysis of healthy donor naive Mos differentiated for 18 h in the presence of BSA-bound PA (red), BSA (blue) or in the absence of BSA (orange). **(A)** Heatmap representation of the log2 vst of transcripts with a log2 FC ≥ 4 and belonging to the GO pathway ‘fatty acid metabolic process’. **(B)** Heatmap representation of the log2 vst of the selected marker of lipid exposition.

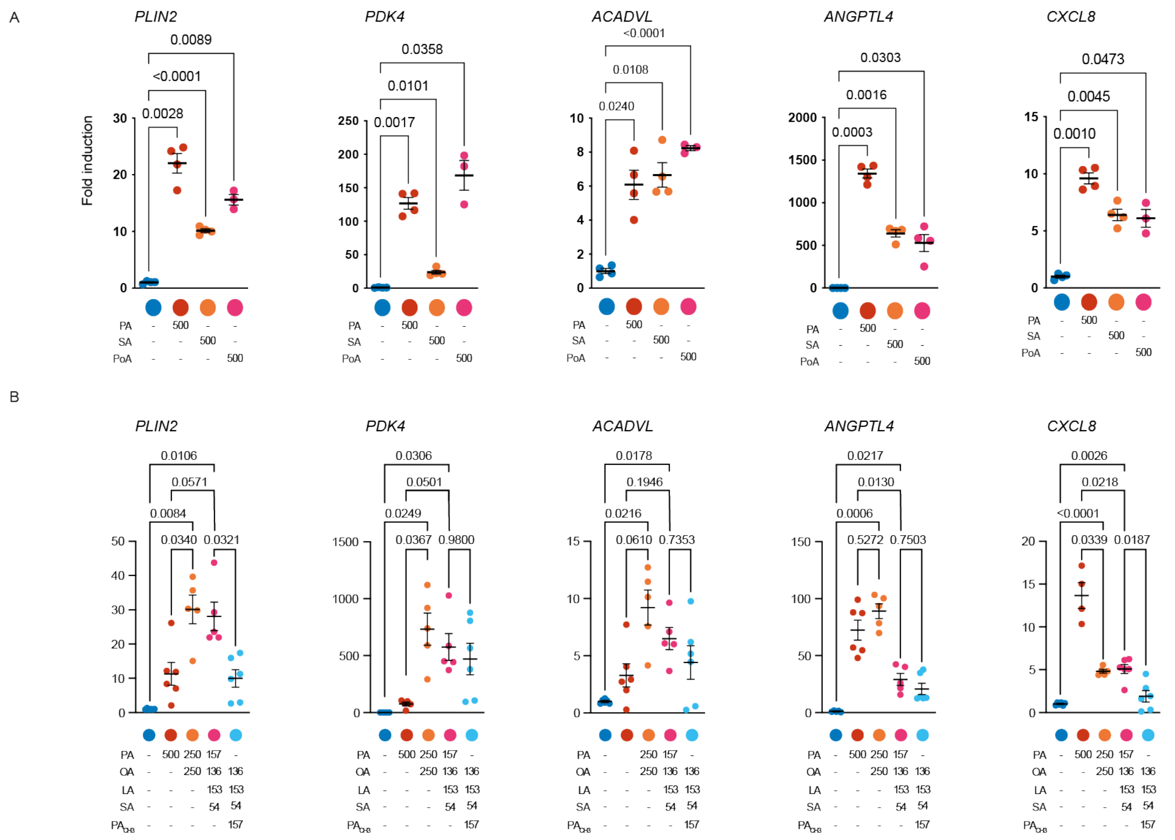

Supplemental Figure 2. FFA alone or in combination induce the expression of *PLIN2, PDK4, ACADVL, ANGPTL4*, and *CXCL8.* (A). RT-qPCR quantification of *PLIN2*, *PDK4, ACADVL, ANGPTL4*, and *CXCL8* in healthy-donor naive Mos treated for 18 h with either BSA only (unbound BSA, blue dots), palmitate (PA-bound BSA, red dots), stearate (SA-bound BSA, orange dots), or palmitoleate (PoA-bound BSA, magenta dots). The values represent the mean (± SEM) of a minimum of independent culture points. p-values were determined by one-way Welch ANOVA tests ($p < 0.0001$, $p = 0.0003$, $p < 0.0001$, $p < 0.0001$, and $p < 0.0001$ for *PLIN2*, *PDK4, ACADVL, CXCL8*, and *ANGPTL4,* respectively) followed by Dunnett's T3 multiple comparison tests (p values are given for BSA vs the indicated group). **(B)** RT-qPCR quantification of *PLIN2*, *PDK4, ACADVL, ANGPTL4*, and *CXCL8* in healthy-donor naive Mos treated for 18 h with either PA (red dots), PA and oleate (OA-bound BSA, orange dots), or a blend of PA, OA, lineate (LA) and SA BSA-bound FA at the indicated concentration (magenta dots) or the same blend where PA is replaced by PA-CH3 (light blue dots). The values represent the mean (± SEM) of independent culture points. P-values were determined by one-way Welch ANOVA tests ($p < 0.0001$, $p = 0.0004$, $p = 0.0006$, $p < 0.0001$, and $p < 0.0001$ for *PLIN2*, *PDK4, ACADVL, CXCL8*, and *ANGPTL4,* respectively) followed by Dunnett's T3 multiple comparison tests. (p values are given for the indicated group)

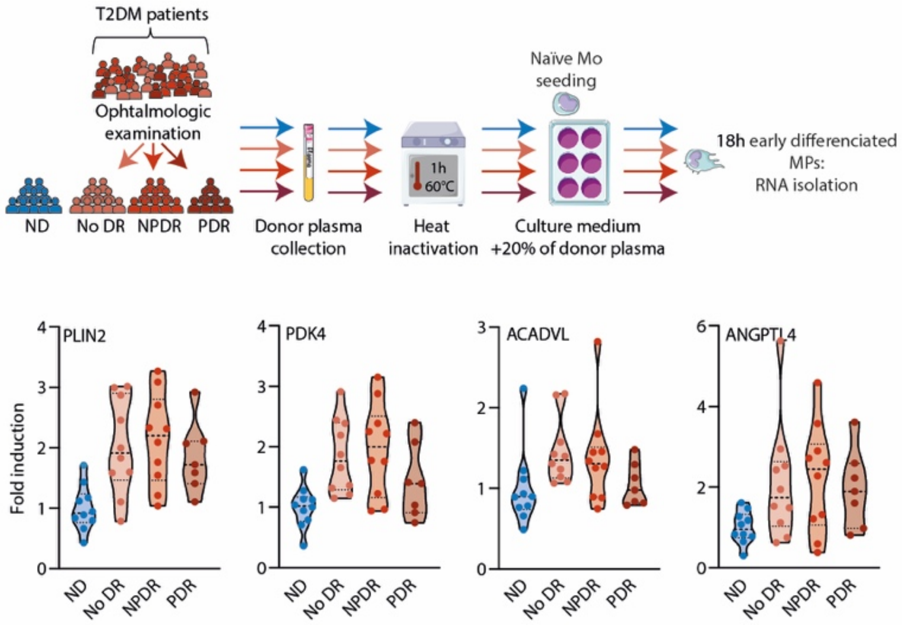

Supplemental Figure 3. Effects of plasma from different groups of patients on lipid-associated genes in MPs. (Upper panel) Schematic representation of donor phenotyping and group attribution, plasma preparation, and naïve Mo treatment. (**Lower panels**) and analyzed by RT-qPCR. (B) Violin plot representation of the relative expression of *PLIN2*, *PDK4*, *ACDVL*, and *ANGPTL4* determined by RT-QPCR in ealthy donor naive Mos treated for 18 h with 20% heat inactivated individual donor plasma (dots) from either ND (n = 10), NO DR (no clinicals signs of Diabetic Retinopathy; n=10), NPDR (non-proliferative DR; n=10) or PDR (proliferative DR; n=7). Dashed lines represent the median and quartiles.

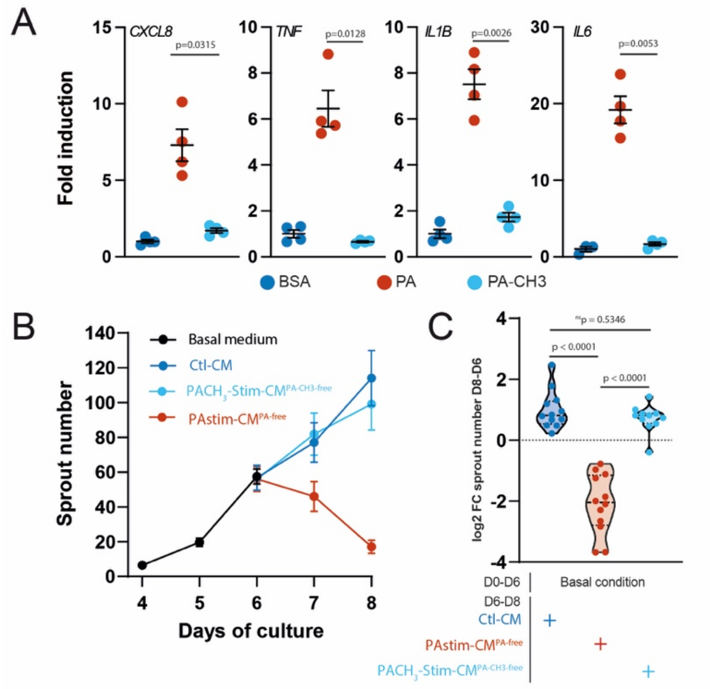

Supplemental Figure 4. PA-CH3 does not trigger *CXCL8*, *TNF*, *IL1β*, or *IL6* expression nor induce vascular sprout degeneration (A) RT-qPCR expression of *CXCL8*, *TNF, IL1*, and *IL6* by in healthy-donor naive Mos treated for 18 h with either BSA only (unbound BSA, blue dots), PA (PA-bound BSA, red dots), or PA-CH3 (PA-CH3-bound BSA, cyan dots). The values represent the mean (± SEM) of independent culture points. p-values were determined by one-way Welch ANOVA tests ($p = 0.0034$, $p = 0.0050$, $p = 0.0005$ and $p = 0.0011$ for *CXCL8*, *TNF, IL1*, and *IL6,* respectively) followed by Dunnett's T3 multiple comparison tests (p values are given for the indicated group). (**B**) Time course representation of the mean (± SEM) sprout number of aortic rings from D4 to D8 treated with Ctl-CM, PAstim-CM$^{\text{PA-Free}}$ or PACH$_3$stim-CM$^{\text{PA-CH3-Free}}$. (**C**) Violin plot representation of the log2 FC in sprout number between paired D6 (before stimulation) and D8 (2 days after stimulation) rings; dots represent individual aortic rings and dashed lines the median and quartiles. p-values were determined by one-way Welch ANOVA tests ($p < 0.0001$) followed by Dunnett's T3 multiple comparison tests.

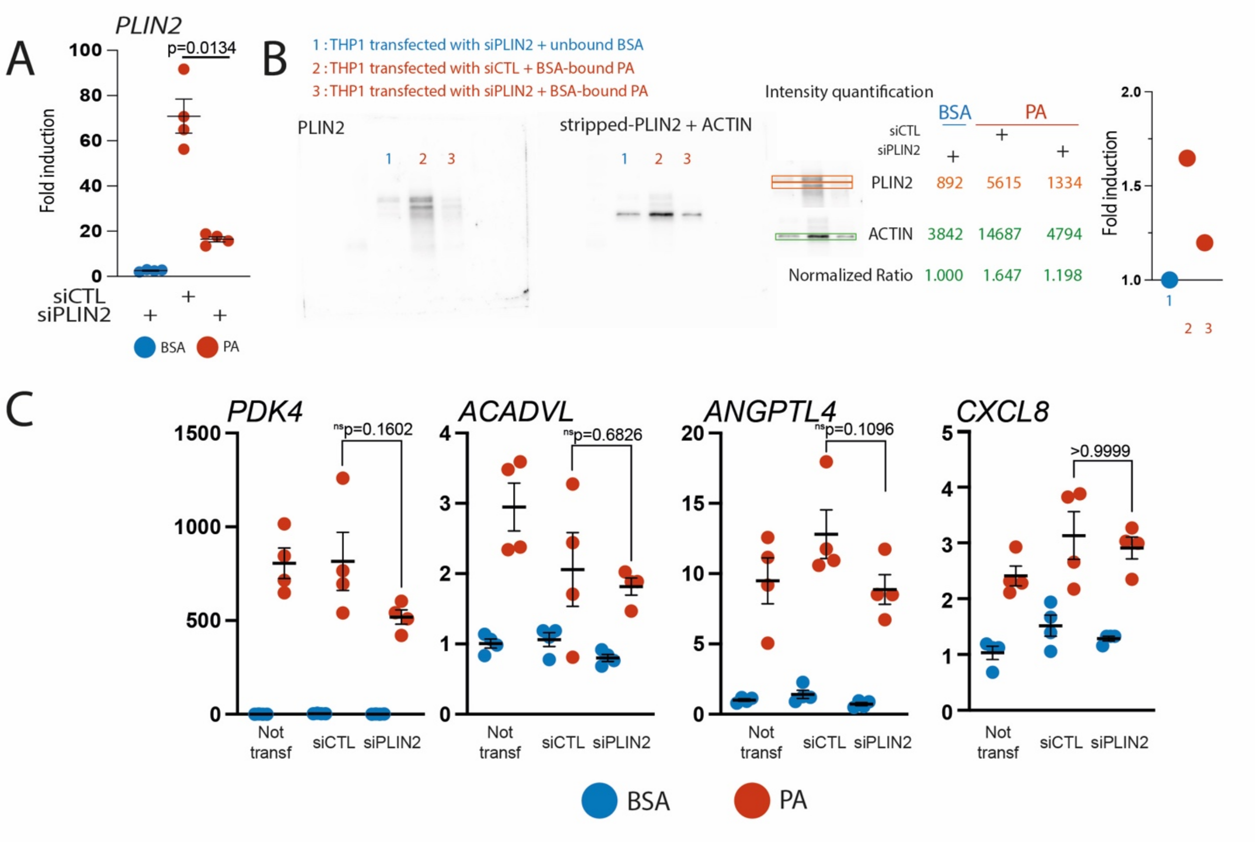

Supplemental Figure 5. ***PDK4, ACADVL, ANGPTL4*, and *CXCL8 are overexpressed in* PLIN2-silenced THP1 cells stimulated with Palmitate. (A)** Scatter plot representation of the RT-qPCR quantification of *PLIN2* in THP1 cells transfected or not with the indicated siRNA and treated 6h later for 18 h with either BSA only (unbound BSA, blue dots) or palmitate (PA-bound BSA, red dots). The values represent the mean (± SEM) of a minimum of n = 4 independent culture points. p-values were determined by one-way Welch ANOVA tests (p = 0.0003) followed by Dunnett's T3 multiple comparison tests (p values are given for PA+siCTL vs PA+siPLIN2) **(B)** Uncropped images of Western Blot analysis of PLIN2 and ACTIN in THP1 cells transfected with siCTL and siPLIN2 and stimulated with BSA or PA (first two left images), the quantitative band analysis (right images) and the relative quantification of PLIN2. **(C)** Scatter plot representation of the RT-qPCR quantification of *PDK4, ACADVL, ANGPTL4*, and *CXCL8* in THP1 cells transfected or not with the indicated siRNA and treated 6h later for 18 h with either BSA only (blue dots) or PA (red dots). The values represent the mean (± SEM) of a minimum of n = 5 independent culture points. p-values were determined by one-way Welch ANOVA tests (p = 0.001, p = 0.0007, p = 0.0001, and p = 0.0004 for *PDK4, ACADVL, ANGPTL4*, and*, CXCL8* respectively) followed by Dunnett's T3 multiple comparison tests (p values are given for PA+siCTL vs PA+siPLIN2).

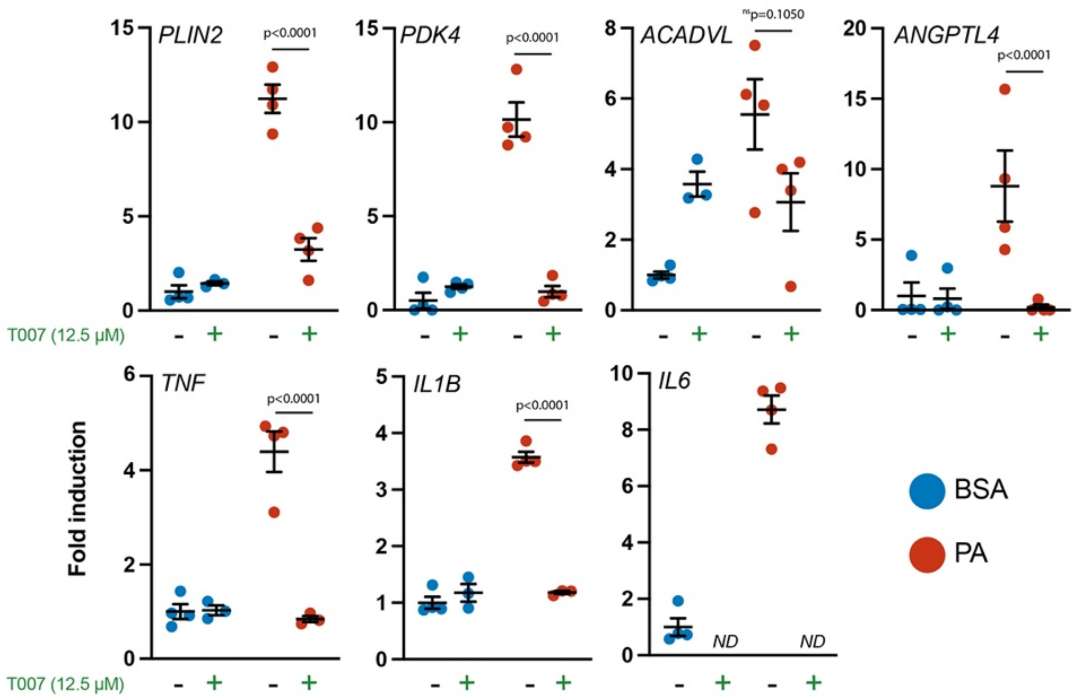

Supplemental Figure 6. Inhibition of PPARγ by T0070907 normalizes the mRNA levels of key markers of Mos exposed to lipids Scatter plot representation of the RT-qPCR quantification of PA-induced transcripts in healthy-donor naive Mos treated for 18 h with either PA (PA-bound BSA, red dots) or BSA only (unbound BSA, blue dots) and the PPARγ antagonist T0070907 (T007) at 12.5 µM. For detected targets, the values represent the mean (± SEM) of n = 3-4 independent culture points; p-values were determined by two-way ANOVA tests. Turkey' multiple comparison tests were used to assess the effect of PA vs that of PA+T007. ND: not detected, no statistical test was performed for *IL6*.

METHODS

Experimental design

The primary goal of this study was to explore the presence of PLIN2$^+$ MPs in the retina of human DR patients. To demonstrate the non-anecdotal presence of PLIN2$^+$ cells in DR retinas, we collected retina from four donors with a known history of DM and compared to one control retina from a non-DM donor. Due to the rarity and considerable cost of human *post-mortem* retinas, we stopped eye collection after demonstrating the presence of PLIN2$^+$ MPs around at least one aneurism in the first 4 patients.

The second goal of this study was to determine the molecular cues that promote the differentiation of Mos into PLIN2$^+$ MPs. We chose to only use naive primary human cells to ensure the translational relevance of our study. Due to the constrains inherent to the collection and isolation of primary human Mos, each stimulation experiment was performed on a single donor (in total, this study involved 69 individual Mo isolations). The viability and purity of the isolations were systematically assessed by a qualified experimenter. A consistent response to PA stimulation (assessed by *PLIN2* upregulation) was prospectively determined as the primary inclusion criterion before subsequent analysis. All donors were found to respond to PA. The fold of induction differed between the donors but was not a subject of the analysis in the present study and thus no donors were excluded from the analysis. One PA preparation was found to not induce a Mo response and experiments using this preparation were excluded from the analysis. In other cases, all PA-solubilization preparations were able to induce the Mo response. A number of PA preparations used in this study were also successfully used in Couturier et al, 2021 (1). Intra-experiment

replicates varied between n = 3 and n = 6, with the number being dependent on the number of Mos isolated from the donor and the number of experimental conditions to be tested. To improve the robustness of the presented data and account for possible RNA degradation during the RNA isolation process, RT-qPCR data points were subjected to agnostic outlier identification using Grubbs' method (alpha = 0.05) in GraphPad Prism 9 (RRID:SCR_002798), and any identified outliers were removed from the subsequent statistical analysis.

For the experiments using human plasma from T2DM and CTL donors, we used a bank established prior to the present study to stimulate naive donor Mos. Compelling preliminary data from 13 plasma samples (two-tailed t test, p = 0.1464) were used to help in determining the optimal sample size. We chose a study design with n = 40 plasma samples (n = 10 control ND plasma and n = 30 T2DM plasma samples evenly distributed between T2DM without DR, NPDR, and PDR). This represented a reasonable sample size operable by a single qualified experimenter and complied with our cell culture laboratory facility and donor Mo availability. It is sufficient to achieve an estimated power of 99.3%, 93.8%, and 79.4% for alphas of 0.05, 0.01, and 0.001, respectively, for ND n = 10 vs T2DM n = 30 plasma samples (Kane SP. Sample Size Calculator. ClinCalc: https://clincalc.com/stats/samplesize.aspx.). No outliers were removed from the analysis. However, for technical reasons, three plasma samples were not used for the stimulation because clots or hemolysis were found at the time of stimulation.

Finally, we designed an alternative version of the classical rat aortic ring to study vasodegeneration for which we have published a comprehensive method paper (2). To prevent substantial variability between samples, statistical analysis was performed on the log2 FC of the sprout numbers between paired D6 (before stimulation) and D8 (2 days after stimulation) rings. Under our conditions, we

routinely observed the log2 FC growth of sprout numbers between paired D6 and D8 rings of ~1.0 (SD ± 0.65) in the control condition. In the present study, we chose to study a biological difference of a 50% decrease (or a 100% increase) in the log2 FC of sprout number and found that seven replicates per group is the minimum to achieve a power of 80% for an alpha set at 0.05.

Post-mortem retina immunofluorescence analysis

Eyes from donors with a known history of diabetes and controls were collected through the Minnesota Lions Eye Bank (Supplemental Table 1). Donor families gave informed consent. Whole eyes were fixed with 4% paraformaldehyde (PFA) before shipping. The retinas were cut in 2-mm diameter punches and stored at -80°C in PFA until use.

Before immunostaining, punches were treated with 0.5% triton X-100 and postfixed with acetone. Punches were incubated with primary antibodies (Supplemental Table 09) for at least two days at 4°C, and then incubated with secondary antibodies at 1/500 for 2 h at room temperature. Nuclei were counterstained with 1/1000 Hoechst. Punches were flat mounted and images were acquired using an inverted Olympus confocal FV1000 (RRID:SCR_016840) microscope or a Yokogawa CQ1 imager (RRID:SCR_023270).

FFA solubilization and medium preparation

Mo were cultured in 'DMEM, no glucose' (ThermoFisher Scientific Cat. #11966025) supplemented with 5 mM glucose, 20 mM mannitol (Merck, RRID:SCR_001287, Cat. #240184), 1 mM pyruvate (ThermoFisher Scientific, RRID:SCR_008452, Cat. #11360070), and 50 unit.ml^{-1} penicillin - 50 µg.ml^{-1} streptomycin (ThermoFisher Scientific, Cat. # 15140122). Different

glucose concentrations (2.5 and 25 mM) were also used for certain specified experiments. Mannitol concentration was adjusted to maintain osmolarity.

To allow FFA solubilization in culture medium, FFA (PA, SA, PoA, OA, LA (Merck (RRID:SCR_001287), Cat. #P0500; #S4751; #P9417; #O1008; #L1376, respectively)) were bound to BSA FFA-free (Merck, Cat. #A8806). FFA were first dissolved in absolute ethanol (EtOH) to obtain a stock solution of 0.1 M and stored at -20°C. This solution was diluted at 1:200 in BSA-culture medium (0.88% w/v of BSA) to obtain the working concentration (500 μM FFA, 0.5 % v/v EtOH; FFA/BSA molar ratio of 3.8). Different FFA concentrations were also used for certain specified experiments. BSA and EtOH concentrations remained unchanged in all settings.

Plasma donor cohort recruitment

Plasma samples from T2DM patients and healthy donors were collected from Mexican volunteer donors at the Institute of Ophthalmology Foundation Conde de Valenciana (Mexico City, Mexico). Collected data included physiological (sex, age, body mass index [BMI], and blood pressure) and biochemical plasma parameters (HbA1c, glucose, total cholesterol, HDLc, LDLc, VLDLc, triglycerides, and creatine). For the T2DM patients, the eye fundus of the worst eye was used to determine DR grading. All donors were Mexican citizens. No other demographic information was collected. The study approval is detailed bellow.

Monocyte isolation and treatment

Human naive Mos were isolated from the peripheral blood of healthy volunteer donors or cytapheresis residuum. Lymphoprep (Stemcell Technologies, RRID:SCR_013642, Cat. #07851) density gradients were used for mononuclear cell isolation and $CD14^+$ Mos were negatively

selected using an EasyStep Human Monocyte Enrichment Kit (Stemcell Technologies, Cat. #19059). Mos were seeded at approximately 470,000 cells.cm^{-1} and naively differentiated for 18 h or treated with FFA. In specified experiments, the effects of various glucose concentrations or PPARα and PPARγ agonists or antagonists were also tested. Treatment started at the time the cells were seeded (t 0h) and lasted until the end of the culture period (t 18h).

Conditioned medium preparation

To obtain CM free of BSA and PA, Mos were first cultured for 18 h and then culture medium was removed. The early differentiated MPs were then cultured for another 24 h in fresh control culture medium. For Aortic ring and HUVEC proliferation CM were concentrated 30x using Amicon Ultra-4 10K Centrifugal Filters (Merck, Cat. #UFC801024).

PLIN2 silencing

THP1 cells (kindly provided by Dr Fitting, C., Institut Pasteur, Paris, France) were FBS-starved overnight and transfected with Lipofectamine RNAiMAX (ThermoFisher Scientific, USA, Cat. # 13778100) for 6 h with 50 nM of a control siRNA (siCTL Silencer® Negative Control No. 2 siRNA (ThermoFisher Scientific, Cat. #AM4613)) or with *PLIN2*-targeting siRNA (ThermoFisher Scientific, siRNA ID s1055, Cat. # 4392420) in Opti-MEM (Thermo Fisher Scientific Cat. #51985034). After the 6 h, transfection medium was removed and THP1 were seeded in NG or NGPA for 18h before RT-qPCR and Western-blot experiments.

Western blotting

THP1 cells were lysed in RIPA lysis buffer (ThermoFisher Scientific, Cat. #89900). Protein concentration was assessed with the Pierce BCA Protein Assay Kit (ThermoFisher Scientific, Cat.

#23227). 20 µg of protein extracts were separated in 4–15% SDS-PAGE precast gels (Bio-Rad Laboratories, RRID:SCR_008426, Cat. #4561086), transferred on a nitrocellulose membrane (Bio-Rad Laboratories, Cat. #1620115) and incubated with PLIN2 (1:1000; Invitrogen Cat# PA5-29099) or β-actin (1:2000; Cat. #8H10D10, Cell Signaling Technology) antibodies. After overnight incubation at 4°C , membranes were incubated with HRP-conjugated secondary anti-rabbit or anti-mouse (1:2500, Cat. #7074s and #7076s, Cell Signaling Technology) antibodies and revealed with Amersham ECL Prime Western Blotting Detection Reagent (Cytiva, USA Cat. # RPN2232). Band intensities were analyzed using ImageJ Analyze Gel tool (Rasband, W.S., ImageJ, U. S. National Institutes of Health, Bethesda, Maryland, USA, https://imagej.nih.gov/ij/, 1997-2018).

HUVEC culture and treatment

Human umbilical vein endothelial cells (HUVECs) were obtained from PromoCell (Germany, Cat #C-12253), and grown no later than passage 10 in EGM™ Endothalial Cell Growth Medium BulletKit™ (Lonza (RRID:SCR_000377, Cat. #CC-3124) medium until stimulation. For proliferation assays, concentrated CM were diluted 1:30 in EGM medium. HUVECs were cultured for 24h, fixed with PFA (ThermoFisher Scientific, Cat. #047392.9M) and stained with 1:1000 Hoechst 34580 (ThermoFisher Scientific, Cat. #H21486) and nuclei counted. For TUNEL assay, HUVEC were starved for 2h and then stimulated with CM (1:4). After 48h, the number of TUNEL positive cells was determined using the In Situ Cell Death Detection Kit, TMR red (Merck, Cat. #12156792910)

Lipidomic analysis

Total lipids were extracted from MPs according to Moilanen and Nikkari (3) and FA composition was determined as previously described (4). The data were processed using EZChrom Elite software from Agilent Technologies (RRID:SCR_013575).

RNA extraction and sequencing and RT-qPCR

RNA extractions were performed using NucleoSpin® RNA isolation kits (Macherey-Nagel GmbH & Co. KG, Germany, Cat. #740990 and #740962). RNA sequencing libraries were constructed from 250 ng total RNA using a modified TruSeq RNA Sample preparation kit protocol. Pass-filtered reads (using Trimmomatic) were mapped using HiSAT2 and aligned to human reference genome GRCh38.95 (5). The count table of the gene features was obtained using HTSeq. Normalization and differential expression analysis values were computed using DESeq2 (6). TPMs were determined using Libinorm using the htseq mode (7).

For RT-qPCR, cDNA was synthesized using the QuantiTect® reverse transcription kit (Qiagen [RRID:SCR_008539], Cat. #205311). qPCR were performed using Power Sybr Green PCR Master Mix (Thermo Fisher Scientific, Cat. #4367659) with the following profile: 10 min at 95°C, followed by a total of 40 two-temperature cycles (15 s at 95°C and 1 min at 60°C). Results are expressed as the fold induction after normalization against housekeeping genes (previously validated by RNAseq data). Primers (Supplemental Table 10).

Multiplex bead immunoassay

Cytokine and growth factor concentrations were measured using the Bio-Plex Pro™ Human Cytokine 27-plex Assay (Bio-Rad Laboratories, Cat. #M500KCAF0Y). Among these

proteins, IL12 and IL13 (not detected under any conditions), as well as IL8 (detected beyond the range of quantification) were not plotted in the graphs.

Aortic ring assay

For the study of vasodegeneration, we designed an alternative version of the classical rat aortic ring to study vasodegeneration for which we have published a comprehensive method paper (2). On day 4, the medium was changed and the daily counting of sprouts was started. On day 6, groups were constituted and treated with 1x dilution of the concentrated CM and the aortic rings cultured for two days. On day 8, some of the aortic rings were fixed with 4% PFA and labeled with anti-collagen IV antibody (Supplementary Table S9) for two days at 4°C. Images were acquired using epifluorescence Leica DM5500 B (RRID:SCR_018896) and inverted confocal Olympus FV1000 microscopes.

Statistical analysis

GraphPad Prism 8 was used for all graphical representations and for all but the RNAseq statistical analysis. For the donor plasma experiments, each individual donor point is represented by a dot and a global distribution profile is shown using truncated violin plots with medians and the quartiles represented as dashed lines. For these experiments, we did not assume normality and the statistical analysis of differences was performed using two-tailed Mann Whitney tests and correlations computed using two-tailed nonparametric Spearman correlations.

For all other experiments, values obtained from individual Mo isolations were assumed to follow a normal distribution, with no assumption of equal variance. Statistical differences between two sample groups were evaluated using two-tailed Welch's t tests and differences between more than

two sample groups were analyzed using one-way Welch ANOVA tests corrected for multiple comparisons by Dunnett T3 test.

When two independent challenges were used (i.e glucose and PA) two-way ANOVA was used, we report individual parameter p-values, as well as parameter interaction p-values, for two-way ANOVA Turkey's post-test was used for multiple comparisons. A p-value (or p-value corrected for multiple comparisons when applicable) of less than 0.05 was considered significant

Study approval

Plasma samples from T2DM patients and healthy donors were collected from Mexican volunteer donors at the Institute of Ophthalmology Foundation Conde de Valenciana (Mexico City, Mexico), with approval from local committees and in accordance with the Declaration of Helsinski : Investigation Committee, Registry 13 CI 09 015 261, Protocol number CI-051-2015. Research Ethics Committee, Registry 13 CEI 09 015 095, Protocol number CEI-2015-11-05 and Biosafety Committee Registry 13 CB 31 050 269, Protocol number CB-051-11-2015. Subjects provided written informed consent prior to participation in the study

Data and materials availability

Experimental materials are available upon request with no restrictions. The RNA-Seq raw fastq files and count files were deposited in the NCBI's Gene Expression Omnibus database (GSE239512).

References:

1. Couturier A, Blot G, Vignaud L, Nanteau C, Slembrouck-Brec A, Fradot V, et al. Reproducing diabetic retinopathy features using newly developed human induced-pluripotent stem cell-derived retinal Muller glial cells. *Glia.* 2021;69(7):1679-93.
2. Blot G, Sartoris TM, Sennlaub F, and Guillonneau X. Modifications to the classical rat aortic ring model to allow vascular degeneration studies. *STAR Protoc.* 2021;2(1):100281.
3. Moilanen T, and Nikkari T. The effect of storage on the fatty acid composition of human serum. *Clin Chim Acta.* 1981;114(1):111-6.
4. Koehrer P, Saab S, Berdeaux O, Isaico R, Gregoire S, Cabaret S, et al. Erythrocyte phospholipid and polyunsaturated fatty acid composition in diabetic retinopathy. *PloS one.* 2014;9(9):e106912.
5. Kim D, Langmead B, and Salzberg SL. HISAT: a fast spliced aligner with low memory requirements. *Nat Methods.* 2015;12(4):357-60.
6. Love MI, Huber W, and Anders S. Moderated estimation of fold change and dispersion for RNA-seq data with DESeq2. *Genome Biology.* 2014;15(12):550.
7. Dyer NP, Shahrezaei V, and Hebenstreit D. LiBiNorm: an htseq-count analogue with improved normalisation of Smart-seq2 data and library preparation diagnostics. *PeerJ.* 2019;7:e6222.
